# Detection of continuous hierarchical heterogeneity by single-cell surface antigen analysis in the prognosis evaluation of acute myeloid leukaemia

**DOI:** 10.1186/s12859-023-05561-0

**Published:** 2023-11-28

**Authors:** Nan Shao, Chenshuo Ren, Tianyuan Hu, Dianbing Wang, Xiaofan Zhu, Min Li, Tao Cheng, Yingchi Zhang, Xian-En Zhang

**Affiliations:** 1 National Laboratory of Biomacromolecules, Institute of Biophysics, Chinese Academy of Sciences, Beijing, 100101 China; 2 State Key Laboratory of Experimental Haematology, National Clinical Research Center for Blood Diseases, Haihe Laboratory of Cell Ecosystem, Institute of Haematology and Blood Diseases Hospital, Chinese Academy of Medical Sciences and Peking Union Medical College, Tianjin, 300020 China; 3 Faculty of Synthetic Biology, University of Shenzhen Institute of Advanced Technology, Shenzhen, 518055 China; 4 University of Chinese Academy of Sciences, Beijing, 100049 China

**Keywords:** Acute myeloid leukaemia, Mass cytometry (CyTOF), AML progression, Trajectory inference, Prognosis evaluation

## Abstract

**Background:**

Acute myeloid leukaemia (AML) is characterised by the malignant accumulation of myeloid progenitors with a high recurrence rate after chemotherapy. Blasts (leukaemia cells) exhibit a complete myeloid differentiation hierarchy hiding a wide range of temporal information from initial to mature clones, including genesis, phenotypic transformation, and cell fate decisions, which might contribute to relapse in AML patients.

**Methods:**

Based on the landscape of AML surface antigens generated by mass cytometry (CyTOF), we combined manifold analysis and principal curve-based trajectory inference algorithm to align myelocytes on a single-linear evolution axis by considering their phenotype continuum that correlated with differentiation order. Backtracking the trajectory from mature clusters located automatically at the terminal, we recurred the molecular dynamics during AML progression and confirmed the evolution stage of single cells. We also designed a ‘dispersive antigens in neighbouring clusters exhibition (DANCE)’ feature selection method to simplify and unify trajectories, which enabled the exploration and comparison of relapse-related traits among 43 paediatric AML bone marrow specimens.

**Results:**

The feasibility of the proposed trajectory analysis method was verified with public datasets. After aligning single cells on the pseudotime axis, primitive clones were recognized precisely from AML blasts, and the expression of the inner molecules before and after drug stimulation was accurately plotted on the trajectory. Applying DANCE to 43 clinical samples with different responses for chemotherapy, we selected 12 antigens as a general panel for myeloblast differentiation performance, and obtain trajectories to those patients. For the trajectories with unified molecular dynamics, CD11c overexpression in the primitive stage indicated a good chemotherapy outcome. Moreover, a later initial peak of stemness heterogeneity tended to be associated with a higher risk of relapse compared with complete remission.

**Conclusions:**

In this study, pseudotime was generated as a new single-cell feature. Minute differences in temporal traits among samples could be exhibited on a trajectory, thus providing a new strategy for predicting AML relapse and monitoring drug responses over time scale.

**Supplementary Information:**

The online version contains supplementary material available at 10.1186/s12859-023-05561-0.

## Background

Despite advancements in clinical therapy, the relapse of acute myeloid leukaemia (AML) occurs in approximately 50% of patients achieving complete remission after initial treatment, thus resulting in high mortality rates owing to a lack of accurate relapse predictors at preliminary diagnosis [1]. Significant heterogeneity generated by disease progression is associated with AML relapse. Leukaemia stem cells (LSCs) harbour various founding mutants that exhibit different surface antigen profiles among individual patients, including CD34^+^/CD38^−^, CD34^−^, and CD38^+^ [2, 3]. During differentiation, mutant clones yield descendants with diverse phenotypes and physiological behaviours [4], and finally form a branching evolution hierarchy with varying degrees of differentiation blockade involving an abundance of malignant progenitors and a few mature myelocytes [5–7]. Previous studies related to the development of the condition have utilized the similar phenotypic progression between abnormal and healthy haematopoiesis to match each leukemic cell to the nearest healthy subpopulation with a constant differentiation stage [8], or have tracked molecular changes of one patient from initial diagnosis to the relapse and post-relapse stage [9]. However, the actual pattern of AML development exhibits a continuous hierarchy with intermediate-state cells between the granular subpopulations [10]; thus, neglecting this hierarchy leads to a biased classification of the intermediate cell using these existing methods. Therefore, to fully describe the hierarchy, we first need to capture specific variations to examine AML blasts at the single-cell level.

In this study, we profiled the single-cell landscape of 36 surface antigens by cytometry time-of-flight (CyTOF, or mass cytometry) [11] for obtaining the phenotype of each cell. We hypothesized that a complete myelocytic lineage exists during proliferation; although this lineage is skewed, the molecular dynamics involved will be similar to that of the healthy hematopoietic process. Using specific algorithms, single cells were automatically aligned according to the phenotype continuum derived from AML progression, which enabled re-constructing the complete myeloid hierarchy using single-cell datasets. We then validated this approach to search for temporal information related to AML chemotherapy outcomes prediction using datasets of paediatric AML patients.

## Methods

### Patient samples

Bone marrow mononuclear cells from 43 paediatric patients with AML were provided by the Chinese Academy of Medical Science, Institute of Haematology, including 17 males and 16 females between the age of 1–14 years old. The cells were suspended in cell-saving liquid (NCM Biotech) and stored in liquid nitrogen. The cells were thawed at 37℃, washed in warm medium [complete RPMI (Hyclone) supplemented with 10% foetal bovine serum (Gibco)], and then cultured for 30 min in medium containing 15 units of DNase I (Lucigen). Nineteen patients achieved complete remission (CR) after chemotherapy treatment, seven showed non-remission (NR), one showed partial remission, seven patients relapsed, six died due to complications, and three did not have complete follow-up information. Detailed information is provided in Additional File 1: Table S1.

### Mass cytometry measurements and data pre-processing

Mass cytometry reagents were supplied by Fluidigm Inc. Lanthanide metal labelling and surface antigen-target antibody staining were performed using MaxPAR antibody conjugation and MaxPAR antibody labelling kit protocols, respectively. Each stained sample was labelled with a unique barcode using Cell-ID 20-Plex Pd Barcoding Kit for mass cytometry detection within a mixture. According to the manufacturer instructions, each mixture dataset was collected with 3 × 10^5^ cells per sample. FCS files created for the mixed barcoded specimens were separated into individual datasets using Debarcoder software (https://www.fluidigm.com/products-services/software), and then each sample was filtered to preserve living myeloid cells according to specific metal signals and biomarkers using Flowjo (version x.0.7) (Additional File 1: Fig. S1). The lanthanide-antibody panel, article numbers, and gating strategy are shown in Additional File 1: Table S2.

### Feasibility test of the trajectory inference method with public datasets

Subsequent analyses were performed using R (version 4.0.0). The workflow for differentiation trajectory analysis was verified using a public dataset with samples SJ03, SJ05, SJ11, and SJ16 reported by Levine et al. [12]. Following the extraction of myeloid cells, 3000 sampled myelocytes were visualized by PhenoGraph [12] and uniform manifold approximation and projection (UMAP) [12] algorithms with default parameter to align the myelocytes according to their differentiation order and phenotypic similarity. The SCORPIUS package [13], a tool for linear trajectory inference that automatically links initial, intermediate, and terminal clusters based on principal curve analysis, was then used to draw a differentiation trajectory on the two-dimensional visualized blast. Based on its position on the trajectory, a single cell was endowed a “pseudotime” value as its degree of maturation, and the hierarchical changes of the tested molecules were visualised in a time-series heatmap.

The early-stage blast was chosen by selecting cells with a pseudotime value < 0.1; these were then visualized on a CD34-CD38 plot by overlapping raw data cells. Furthermore, five reported hierarchical signal pathway responses, G-CSF → pSTAT3, SCF → pAkt, G-CSF → pSTAT5, Flt3-L → pAkt, and IL-10 → pSTAT3, were respectively visualized with an integrative presentation of temporal expression changes before and after stimulation. In brief, temporal changes of each inner molecule in two situations were fitted by locally weighted regression (LOESS) and integrated into a time-expression coordinate system.

### Dispersive antigens in neighbouring clusters exhibition (DANCE) feature selection strategy

We optimized the trajectory inferred by the proposed pipeline with the myeloid blasts of the 43 clinical samples described above. Following data collection and pre-processing, 8000 cells in each sample were selected at random and 20 surface antigens involved in normal hematopoietic processes were used for differentiation-related feature selection. For each sample, molecules with positive expression (raw data value > 10 in up to 5% cells) were selected. These data were then subjected to hyperbolic arcsine (arcsinh) transformation and clustered using Phenograph for DANCE selection. We created a distance matrix representing the Euclidean distance between each of the two clusters and selected the three shortest-distance neighbouring clones to every cluster. Thus, each cluster would comprise a four-neighbouring group. In every group, the expression levels of single molecules fluctuating on different scales were calculated according to the variance of an antigen in each group; if the variance was < 0.5 in up to 25% of groups, the surface antigen would be preserved. After counting the occurrence of the DANCE-selected antigens among the 43 patients, we identified eight antigens that were selected in the majority of samples, which were combined with the constant differentiation markers CD45, CD38, CD11b, and CD34 to obtain a final antibody combination, which we refer to as the 12-antigen differentiation panel. The new panel was then used to reconstruct the trajectory for each sample. Next, the CellAlign algorithm [13] was adopted to test the correct direction of the trajectories by the normalized dissimilarity value. A typical differentiation trajectory (favourable model fitting and a CD45^high^/CD11b^+^ clone as the mature terminal landmark) was applied as a reference to determine the direction of query one for determining the trajectory direction. The correct direction of the query trajectory generated a lower normalized dissimilarity value.

### Misaligned expression and stemness heterogeneity temporal distribution

The 32 samples exhibiting excellent trajectory fitting and with complete follow-up information available after chemotherapy were selected for further misaligned expression analysis (Additional File 1: Fig. S2). After obtaining pseudotime values via the 12-antigen differentiation panel, the cells were ranked linearly in temporal order and the single-cell expression level of CD11c was plotted along the vertical coordinates. Regarding cells with a pseudotime value < 0.6 as primitive clones and others in the mature stage, the mean expression levels of the two stages in each sample were calculated. The samples were then divided into four groups according to CD11c expression and response/relapse status. The CD11c expression levels in the two stages among the four groups were then compared and integrated into two boxplots and used t-test for significance test.

To explain the temporal features of specific antigen combinations related to AML relapse, we selected 26 samples with a lower mean dissimilarity value than the others (threshold < 0.25, Additional File 1: Fig. S2). After obtaining single-cell pseudotime values via trajectory inference of the 12-antigen differentiation panel, 15 reported stemness-related antigens were used to re-cluster the blasts and then stemness clusters were generated for each cell. According to the differentiation pseudotime of single cells, the trajectory was separated into 10 components on average. The stemness heterogeneity of each time component was then obtained using the population count and type of each contained stemness cluster. In brief, the formula for calculating the stemness heterogeneity index was based on Shannon entropy [H(x) = − ∑p(x) × logp(x)], which was calculated as [H(x) = − ∑ (N_*i*_/N_total_) × log (N_*i*_/N_total_)], where N_*i*_ is the cell number of cluster *i* in a given time period and N_total_ is the count of test cells along the complete trajectory. The stemness heterogeneity index of each time component was then plotted as a histogram with temporal order on the horizontal axis and the dynamics were fitted by LOESS regression. After detecting the time point of the appearance of the first peak in each sample, we integrated the peak time location in 14 patients with CR, 7 with NR, and 5 with relapse.

### Analysis of 10X genomics single-cell RNA-sequencing (scRNA-seq) data

Raw scRNA-seq datasets in FASTQ format were supplied by Ruijin Hospital affiliated to Shanghai Jiao Tong University School of Medicine derived from seven specimens of adult AML bone marrow, including four patients with relapse and three who achieved CR after initial chemotherapy treatment. Data alignment and pre-processing were performed according to a previous study [14]. After visualisation using Phenograph and UMAP, specific molecule-expression clusters were used to determine specific cell types, including CD3D (T lymphocytes), IGJ (B lymphocytes), and CA1 (erythrocytes). Myeloid blasts were selected and re-arranged in the UMAP space according to a reported monocyte differentiation-related panel [15]. The trajectory was then inferred via SCORPIUS and the expression patterns of molecules in mature myeloid blasts (FCN1 and CD14), monocytes (LYZ), abnormal monocytes (PRTN3), and CD34^+^/CD117^+^ clones (CRIP1 and NPW) were determined along with the differentiation ranking of the sample.

Molecules involved in the 17-gene stemness score assessment [16] were applied as parameters of the 17-gene stemness heterogeneity distribution; the index was calculated by CyTOF. Finally, the 17-gene stemness score and temporal dynamics of each sample were used to test the results of stemness heterogeneity.

## Results

### Continuous trajectory inference and LSC identification

LSCs are considered to be responsible for drug resistance, although their identification has long been hindered by the surrounding complex blood system that contains cells with different phenotypes. Here, we tracked a complete myeloid lineage to identify populations from the initial, intermediate, and mature differentiation stages [15]. The inferred trajectory was drawn on the UMAP reduced space that preserves the global similarity of AML cells, and the evolution trail was constructed by linking the principal single-cell alignment; CD11b^+^/CD45^high^ blasts, which are generally regarded as mature myelocytes [17], were automatically arranged at the terminus as a confirmed landmark. We assumed that backtracking the trajectory from mature populations would reveal the initial clones, enabling the entire dynamics of molecules to be described along the trajectory. To verify the feasibility of this strategy, we adopted public datasets and Phenograph algorithm published by Levine et al., which robustly perform cell clustering and recognise primitive and mature clones of AML with both surface antigens and inner signal pathway molecules, and attempted to build a surface antigen-based differentiation trajectory to obtain the same results as found in the literature.

**Fig. 1:**
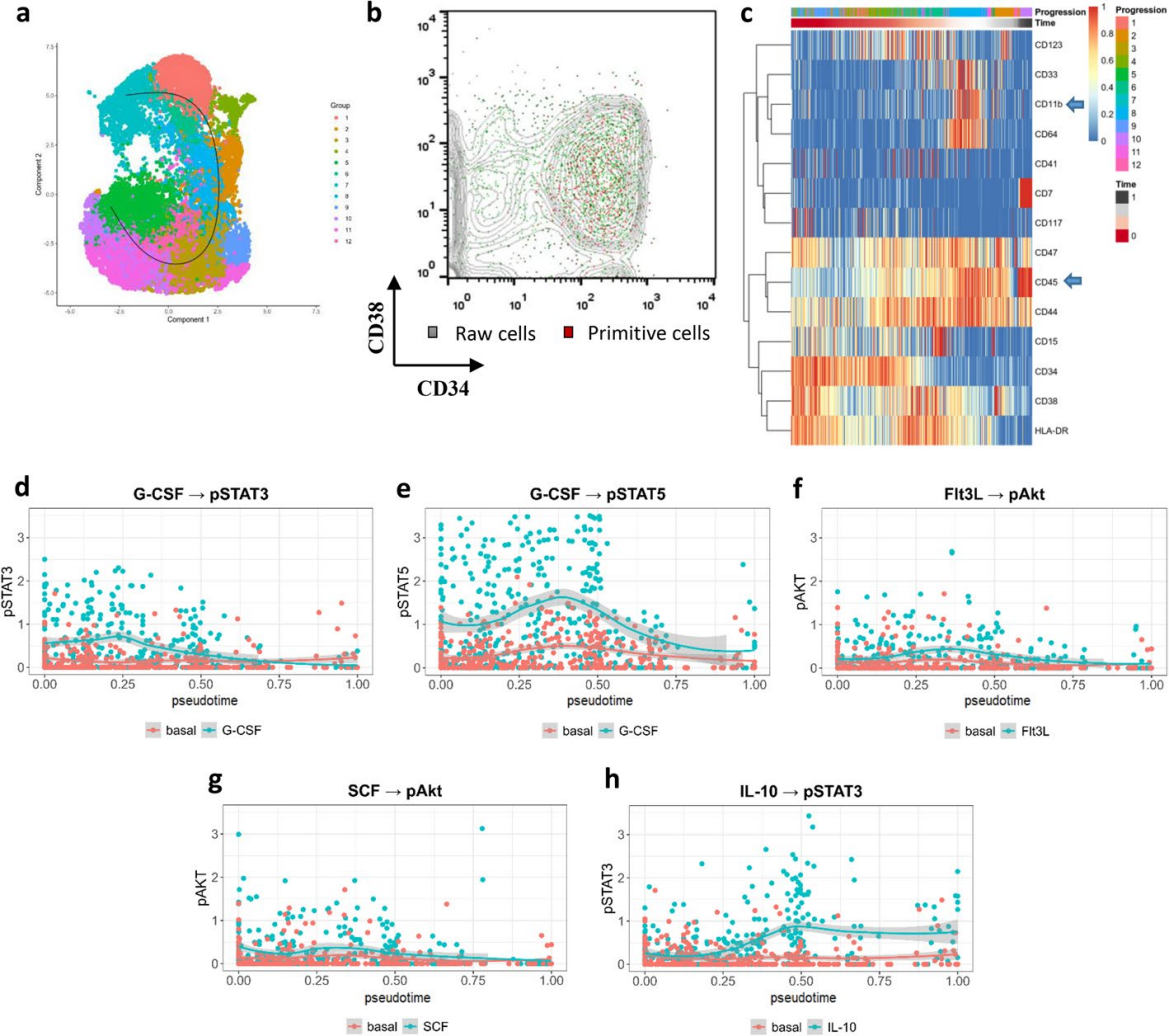
Recognition of CD34^+^CD38^−^ LSC clones using differentiation trajectory and changes in signal molecules in time-series before and after cytokine induction. **a** Differentiation trajectory inference in SJ03; **b** primitive cells emphasized in red with CD34^+^CD38^−^ phenotype, cells with 0.1 < psudotime < 0.8 is labelled by green; **c** time-series heatmap of the reported penal in which mature blasts express CD11b^+^CD45^high^ (blue arrow); **d**, **e** pSTAT3 and pSTAT5 expression increases in the early stage of G-CSF induction; **f**, **g** Akt showed a slight response to the effect of Flt3L and SCF; **h** Increased expression of pSTAT3 after IL-10 induction was observed in the intermediate and late stages

In sample SJ03, the myeloid cells were spread along the UMAP space to present a phenotypic continuum, and then each cell was allocated a pseudotime value by the SCORPIUS algorithm to reflect its degree of maturity (Fig. 1a). Next, clones with a pseudotime < 0.1 were selected as primitive blasts that display a CD34^+^ phenotype, which shared a similar but narrow range in two-antigen expression coordinates as the results in Levine et al. The subpopulation with the similar range in the CD34/CD38 coordinate system has the pseudotime < 0.8 (Fig. 1b). Instead of classifying leukocytes into granular states as published, a progressive heatmap was produced to profile the temporal changes of single-cell molecules along the continuous hierarchy. For example, the HLA-DR expression level was higher in the middle phase, whereas CD64 expression was only present in a few mature cells and CD117-positive cells were mostly found during the middle stages of AML progression (Fig. 1c).

This strategy created a pseudotime value for not only single cells but also for all tested molecules expressed on individual cells. Published results relating to the signal responses to drugs included the following significant patterns: G-CSF → pSTAT3, SCF → pAkt, G-CSF → pSTAT5, Flt3-L → pAkt, and IL-10 → pSTAT3, which were also evident on the trajectory of surface molecules described herein. The continuous temporal hierarchy of sample SJ03 showed that pSTAT3 and pSTAT5 expression was upregulated in the early stages via G-CSF induction (Fig. 1d, e), pAkt showed a slight response to the effect of Flt3L and SCF (Fig. 1f, g), and pSTAT3 expression increased during the intermediate and late stages after IL-10 induction (Fig. 1h).

**Fig. 2:**
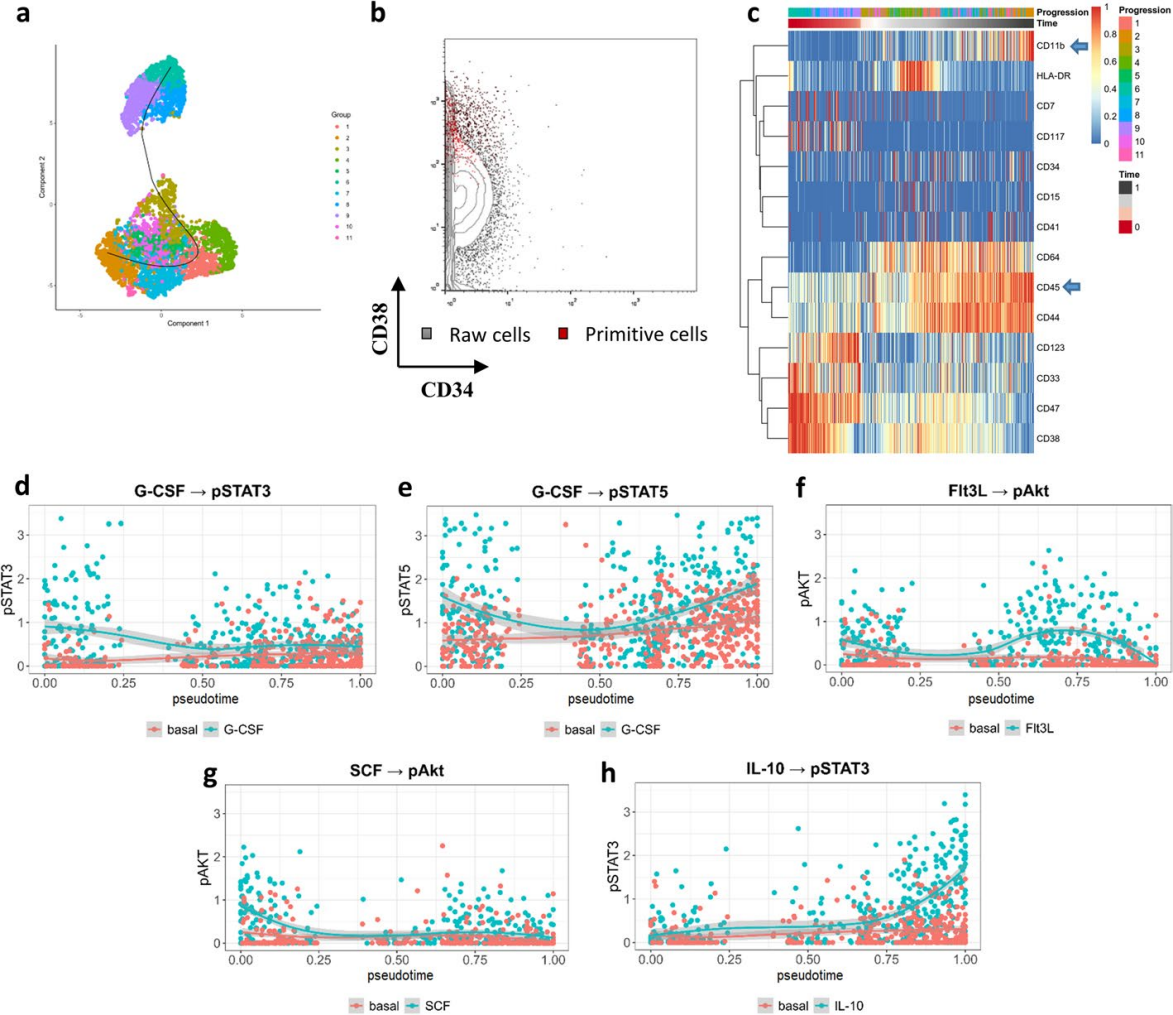
Differentiation trajectory analysis in samples with untypical LSC blast. **a** Differentiation trajectory inference in SJ11; **b** Primitive cells emphasized in red with CD38^+^ phenotype; **c** Time-series heatmap of the reported panel in which mature blasts express CD11b^+^CD45^high^ (blue arrow); **d** and **g** pSTAT3 and pAkt in primitive clones showed slightly unregulated responses to G-CSF and SCF; **e** overall increase of pSTAT5 expression after G-CSF induction; **f**, **h** pAkt and pSTAT3 in mature blasts upregulated with the effect of Flt3L and IL-10

Ignoring the plastic phenotype of LSCs with various genetic backgrounds and phenotypes, our workflow efficiently dealt with samples in which atypical initial clones exhibited a CD34^–^ or CD38^+^ phenotype. In sample SJ11, the starting clones were labelled with CD34^–^/CD38^+^, but on a smaller scale than that described in the original report (Fig. 2a, b). According to the time-series heatmap of this sample, CD11b^+^/CD45^high^ blasts occupied the end of the trajectory, CD117 and CD33 expression was upregulated in the early stage, and CD64 expression increased in mature cells (Fig. 2c). The inner signal molecules also showed behaviours similar to those reported in the literature. In primitive clones, pSTAT3 and pAkt showed slightly unregulated responses to G-CSF and SCF stimulation (Fig. 2d, g), in which G-CSF stimulated an overall increase of pSTAT5 (Fig. 2e), whereas pAkt and pSTAT3 expression levels increased in mature blasts in response to Flt3L and IL-10 stimulation (Fig. 2f, h). The expression behaviours of other samples on the differentiation timeline are shown in Additional File 1: Figs. S2 and S3, suggesting that our methodology was accurate and thus feasible for analysing the progression of AML development.

### Selection of differentiation-related features using clinical samples

Using surface antigen panel in Levine's work, we verified that our continuous time-series analysis strategy described herein recurred the published granular progression results, including primitive subpopulation recognition and inner signal change during AML development. Even though the strategy could deal with individual sample, the panel in the reference used for healthy blood classification to generate metacluster (MC) may not be suitable for our workflow, which directly build trajectory based on AML blast. To efficiently reflect the actual progression process of AML, we designed a feature selection method to extract surface antigen panel and simplify the myeloid differentiation trajectory with more convergency on a single trail created by SCORPIUS.

**Fig. 3:**
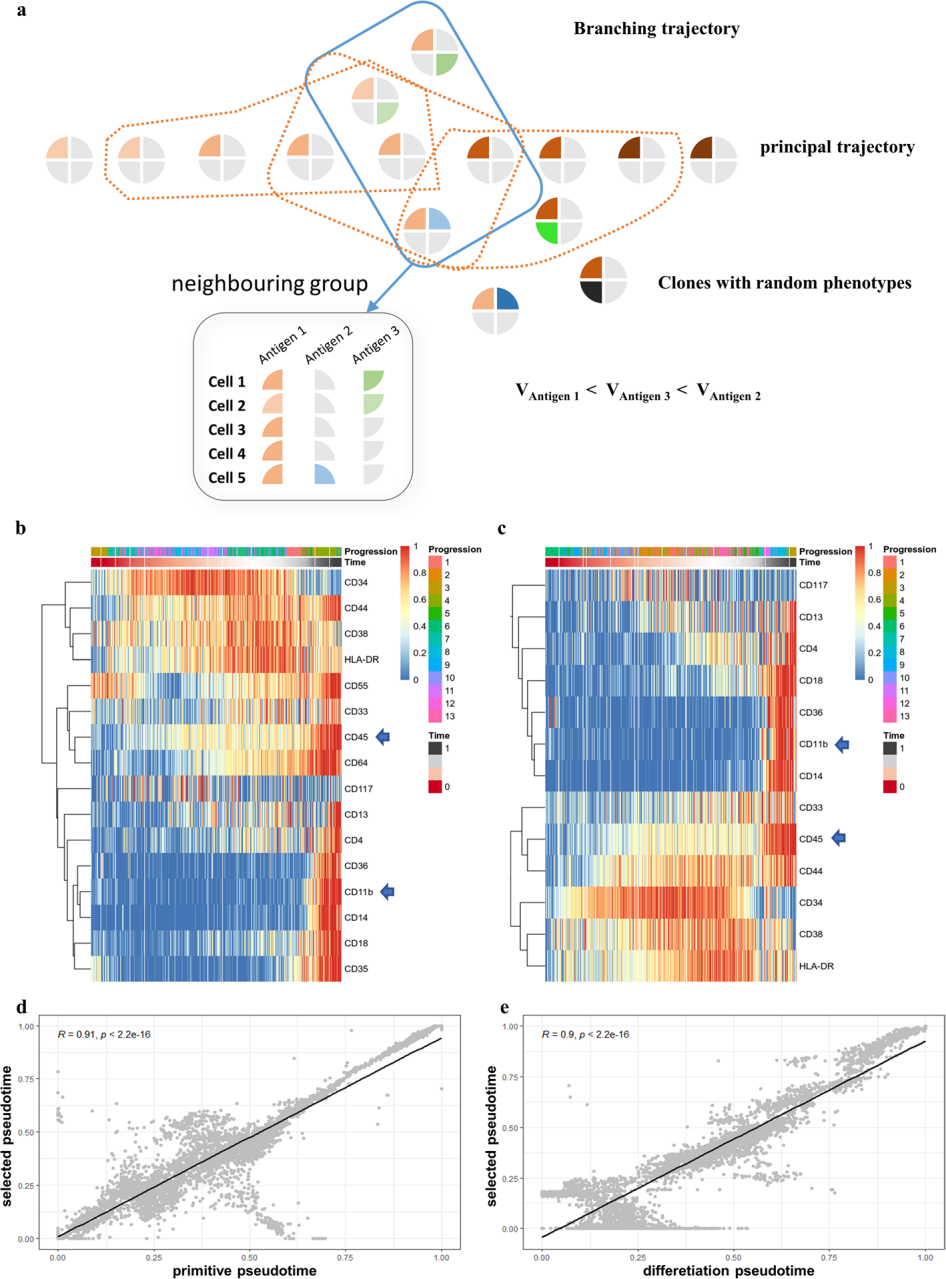
Differentiation conservative feature selection strategy and its application in sample no. 570407. **a** “Dispersive antigens in neighbouring clusters exhibition (DANCE)” feature selection strategy: AML branching evolution model is generated by excess surface antigens. A group of neighbouring clusters (blue frame) was assembled by Euclidean distance and each molecule in the group (grey frame) presented with diverse variance (represented by V). There are several neighbouring groups (orange frames) generated for differentiation feature counting; **b** time-series heatmap derived from 16 markers with a higher expression rate identified each cell with a primitive pseudotime; **c** time-series heatmap derived from the panel after DANCE selection and identified each cell with a pseudotime based on selected panel; **d** pseudotime correlation between primitive and selected panel-inferred trajectory (R = 0.91); **e** pseudotime correlation between feature selected and final panel-derived from the DANCE panel of 43 clinical samples (R = 0.90)

Since there a similar molecular dynamics in haematopoiesis of normal and diseased cells, we utilized 20 surface antigens expressed during normal myeloid differentiation as an alternative selection panel [18] (Additional File 1: Table S2) for the 43 paediatric AML samples. The AML evolution trajectory for redundant features generally presents a branching pattern, including node markers that determine the cell fates and divergent markers expressed in certain branching trails [19, 20]. In addition, a few molecules with low expression ratios might have no function and exist randomly in the blasts. To exclude these three types of molecules, we designed the DANCE method for the selection of differentiation-related antigens (Fig. 3a).

Considering sample 570,407 for example, of the 20 surface antigen candidates, 16 with an expression ratio > 5% in AML blasts were firstly selected (Fig. 3b). The remaining features were then filtered by DANCE, which preserved 13 antigens that retained CD34, CD38, CD11b, and CD45 (Fig. 3c). The single-cell pseudotimes of the two selection steps were generated by SCORPIUS and compared by correlation plots, which indicated no changes in the cell stages after DANCE selection (R = 0.91) (Fig. 3d). After applying DANCE to the samples of all 43 patients, a new panel was obtained for each sample for subsequent statistical analyses (Additional File 1: Table S3). We eventually determined eight antigens, including CD64 (25/43), CD117 (27/43), HLA-DR (25/43), CD33 (35/43), CD44 (35/43), CD18 (30/43), CD4 (30/43), and CD13 (28/43), that were expressed in the majority of patients. These were then combined with the mature (CD11b and CD45) and primitive (CD34 and CD38) biomarkers to constitute a novel panel containing 12 surface antigens (referred to as the 12-antigen differentiation panel). The pseudotime correlation between the selected and final panel of sample 570,407 is shown in Fig. 3e, thus confirming that there were no major changes to the pseudotime point of cells after DANCE selection and determination of the final panel (R = 0.9).

**Fig. 4:**
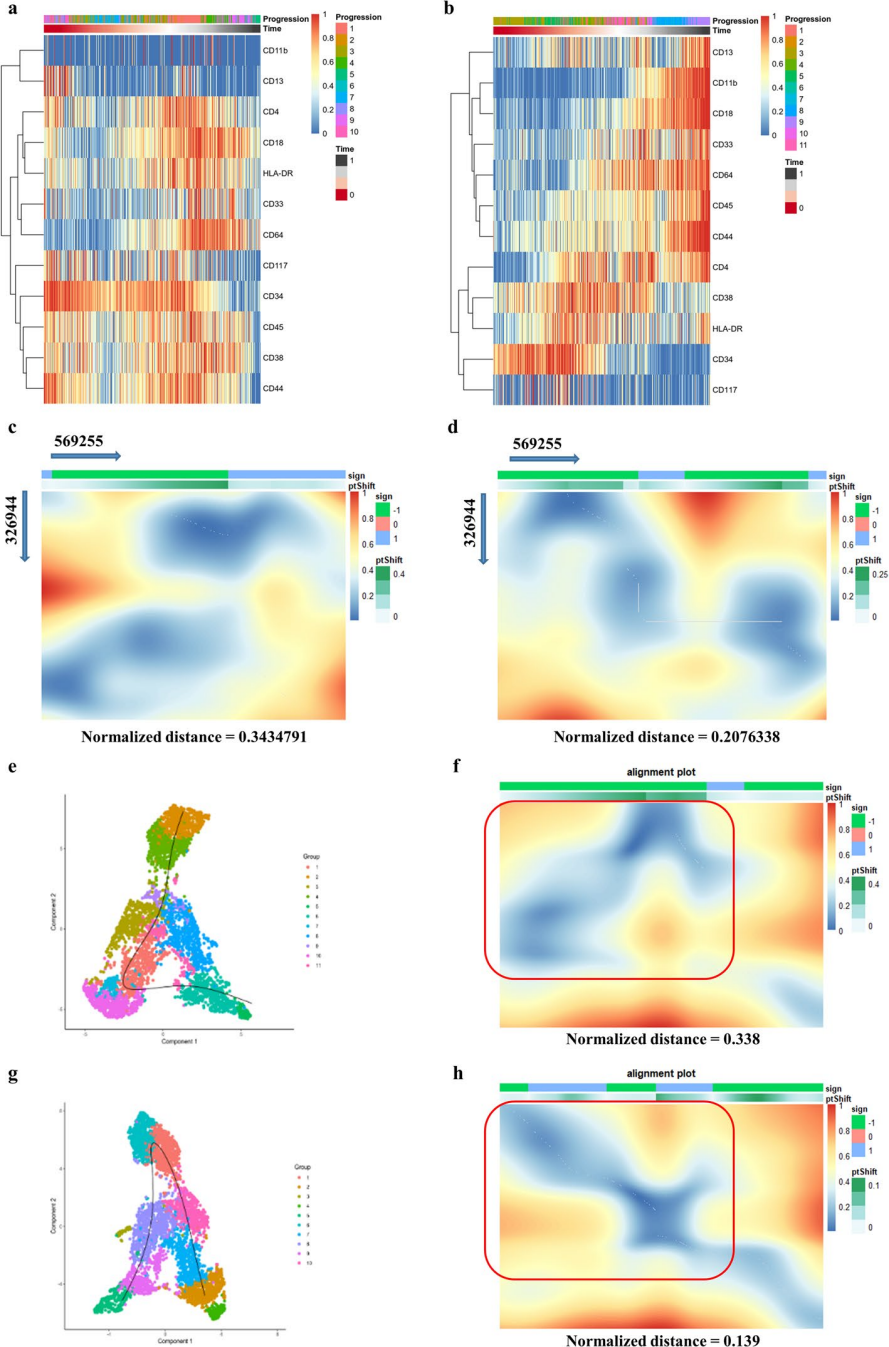
Differentiation orientation pinpoint with the reference of typical trajectory. **a** Time-series heatmap of sample no. 569255 could not pinpoint the trajectory direction without CD45^high^/CD11b^+^ cluster landmark; **b** time-series heatmap of sample no. 326944 (trajectory with typical mature blast at right terminal) as reference trajectory; **c** dissimilarity heatmap of sample no. 326944 and reverse sample no.569255 trajectory; **d** dissimilarity heatmap of sample no. 326944 and correct sample no.569255 trajectory; **e**, **f** trajectory inference with local reverse did not match with the sample on dissimilarity matrix. Red frame emphasized the error-directed local; **g**, **h** correct trajectory direction with a diagonal presentation. Red frame emphasized the local with correct direction

After feature selection, every sample would exhibit a differentiation trajectory with a similar 12-antigen dynamic pattern. We then used the CellAlign algorithm to compare the global dynamics of each pair of trajectories and measured the normalized distance as their dissimilarity. In general, a pair of similar trajectories produces a lower normalized distance and diagonal alignment in a dissimilarity matrix. According to the distance value and matrix of every pairwise trajectory, CellAlign was able to estimate the correct direction of a query trail. To determine the optimal alignment of sample 569,255, which lacked CD11b^+^/CD45^high^ cell blasts in two terminals of the trajectory showed in Fig. 4a, we compared this sample with the reference sample 326,944, which involved a conventional terminus (CD11b^+^CD45^high^ located at the right of trajectory heatmap with largest pseudotime) and expected expansion in UMAP plot (Fig. 4b). After attempting to adjust the direction, the correct cell rank of sample 569,255 matched that of sample 326,944 with a low normalized distance, ranging from 0.343 to 0.208, indicating an alignment closer to the diagonal in the dissimilarity matrix (Fig. 4c, d). CellAlign was also used to determine the local trajectory. The primitive stage of sample 274,360 presented an error direction under the default parameters of SCORPIUS, which was unmatched to the reference sample 296,083 with a normalized distance of 0.338 (Fig. 4e, f). By changing the relative parameters, the early trajectory showed an inverse fit, thus obtaining a dissimilar diagonal line with a lower dissimilarity value (0.139) (Fig. 4g, h). Therefore, the 12-antigen differentiation panel revealed the commonality of AML evolution patterns, demonstrating its potential utility for comparing temporal traits between patients.

### Misaligned expression and the evaluation of prognosis

Asynchronous expression implies that early and mature antigens are co-expressed when detected by flow cytometry. Conventional detection methods in bulk cells generally consider progenitor molecules as a reference; hence, their occurrence with late-stage markers such as the CD117^+^/CD15^+^ phenotype, might predict clinical outcomes [21]. However, flow cytometry cannot separately reflect molecular temporal behaviour directly, and the uncertain phenotype makes it difficult to identify the precise state of a molecule (i.e., CD34 for initial clones). Instead of analysing asynchronous expression according to the expression of such relatively dubious temporal markers, we analysed the dynamics of every single molecule on the trajectory and revealed their typical or misaligned expression stage among the 32 clinical samples with complete prognosis (follow-up) information and good trajectory fitting (Additional File 1: Fig. S4).

**Fig. 5:**
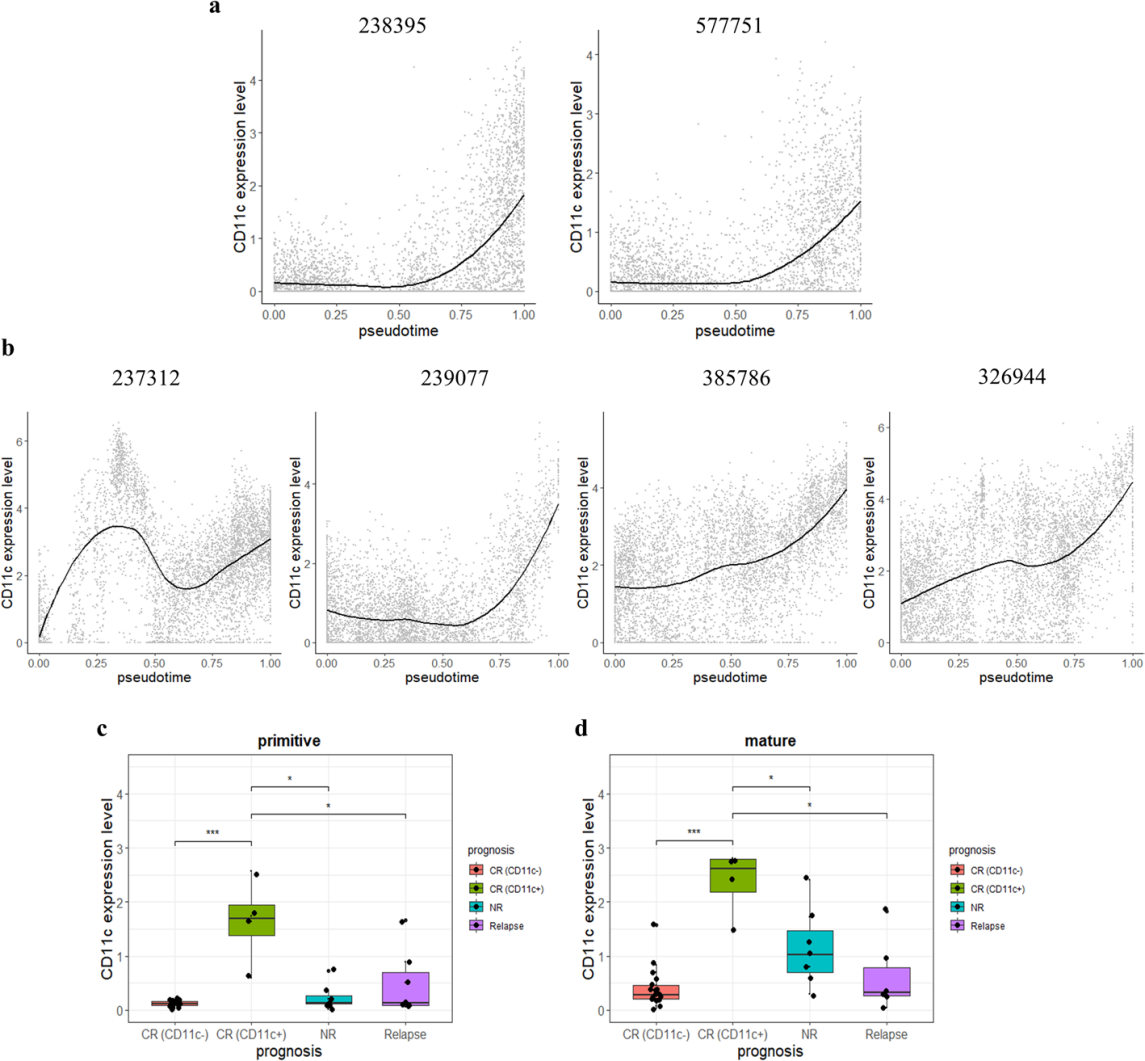
Two CD11c temporal expression patterns and the statistic of CD11c abundance in primitive and mature stages of samples. **a** Expression behavior of CD11c on the trajectory in most patients; **b** CD11c expression was high in the primitive stage of 4 samples with complete-remission outcome; **c** CD11c abundance in different prognosis types in the early stage; **d** CD11c abundance in different prognosis types in the late stage

On the differentiation trajectories constructed using the 12-antigen differentiation panel, the dynamics of other tested molecules (Additional File 1: Table S2) could be ascertained along the pseudotime axis. Next, we observed different behaviours of CD11c among patient samples, which is a molecule that is commonly expressed on the surface of dendritic cells [22], monocytes [23], and B cells [24]. Typically, CD11c was significantly more highly expressed at the end of the trajectory than at the early stage (Fig. 5a). However, four patients with CR, one with NR, and one with relapse had aberrantly high levels of CD11c in the primitive stage (Fig. 5b shows the results for the four CR samples). After identifying each specimen’s expression of CD11c in the primitive (pseudotime < 0.6) and mature stages, we separated the 32 samples into the following groups: complete remission with CD11c^–^ in the early state [CR (CD11c^−^), n = 14], complete remission with CD11c^+^ in the early state [CR (CD11c^+^), n = 4], NR (n = 7), and relapse (n = 7). Statistical analysis was used to investigate the expression levels of CD11c in the two stages of the four groups: the mean expression levels were higher in both stages of the CR (CD11c^+^) group compared with those of the other three groups, with the greatest difference in comparison with the CR (CD11c^–^) group (*p* < 0.001); the temporal changes in most NR and relapse patients were significantly (*p* < 0.05) lower than those in the CR (CD11c^+^) group in the two states (Fig. 5c, d). As shown in Table 1, significantly higher levels were detected in NR samples at their mature stage when compared with those of the CR group (CD11c^–^) (*p* = 0.0033). In contrast to the CR (CD11c^+^) group, which showed significant improvement in CD11c expression in the two stages compared with that of the CR (CD11c^–^), the NR group exhibited a higher CD11c level only in the mature stage. Of the six patients with high CD11c levels in the primitive stage, four achieved CR, thus indicating the potential prediction of a good prognosis in AML diagnosis. In addition, most patients with poor clinical outcomes had slightly higher expression levels of CD11c during the primitive stages as compared with those in the CR (CD11c^+^) group.

**Table 1:** Differences in the expression of CD11c in four groups (*p* value)

	Primitive				Mature			
	CR (CD11c^−^)	CR (CD11c^+^)	NR	Relapse	CR (CD11c^−^)	CR (CD11c^+^)	NR	Relapse
CR (CD11c^−^)		< .001	0.23	0.66		< .001	0.0033	0.61
CR (CD11c^+^)	< .001		0.012	0.019	< .001		0.024	0.019
NR	0.23	0.012		0.95	0.0033	0.024		0.18
Relapse	0.66	0.019	0.95		0.61	0.019	0.18	

### Distribution of stemness heterogeneity along the differentiation trajectory

Before further evaluating the 12-antigen differentiation trajectory, we selected samples with highly similar differentiation patterns for the comparison of temporal features. According to the distribution of dissimilarity values between each sample with the others, we identified 26 (CR, n = 14; NR, n = 7; relapse, n = 5) with a lower mean dissimilarity value (< 0.25, Additional File 1: Fig. S4) to ensure a consistent temporal molecular behaviour for analysis.

Several antigens and transcription factors affect the function and targeting of the initial clones, thus supporting therapeutics selection and prognosis evaluation. However, existing stem cell-related markers exhibit variability in both type and abundance among different patients, thus generating uncertain combinations that make it difficult to determine the status of LSC blasts by specific biomarkers. Instead of attempting to identify a non-specific LSC blast, we focused on stemness as a trait to investigate the proliferation and self-renewal capability of single cells along with the stemness temporal dynamics during differentiation.

**Fig. 6:**
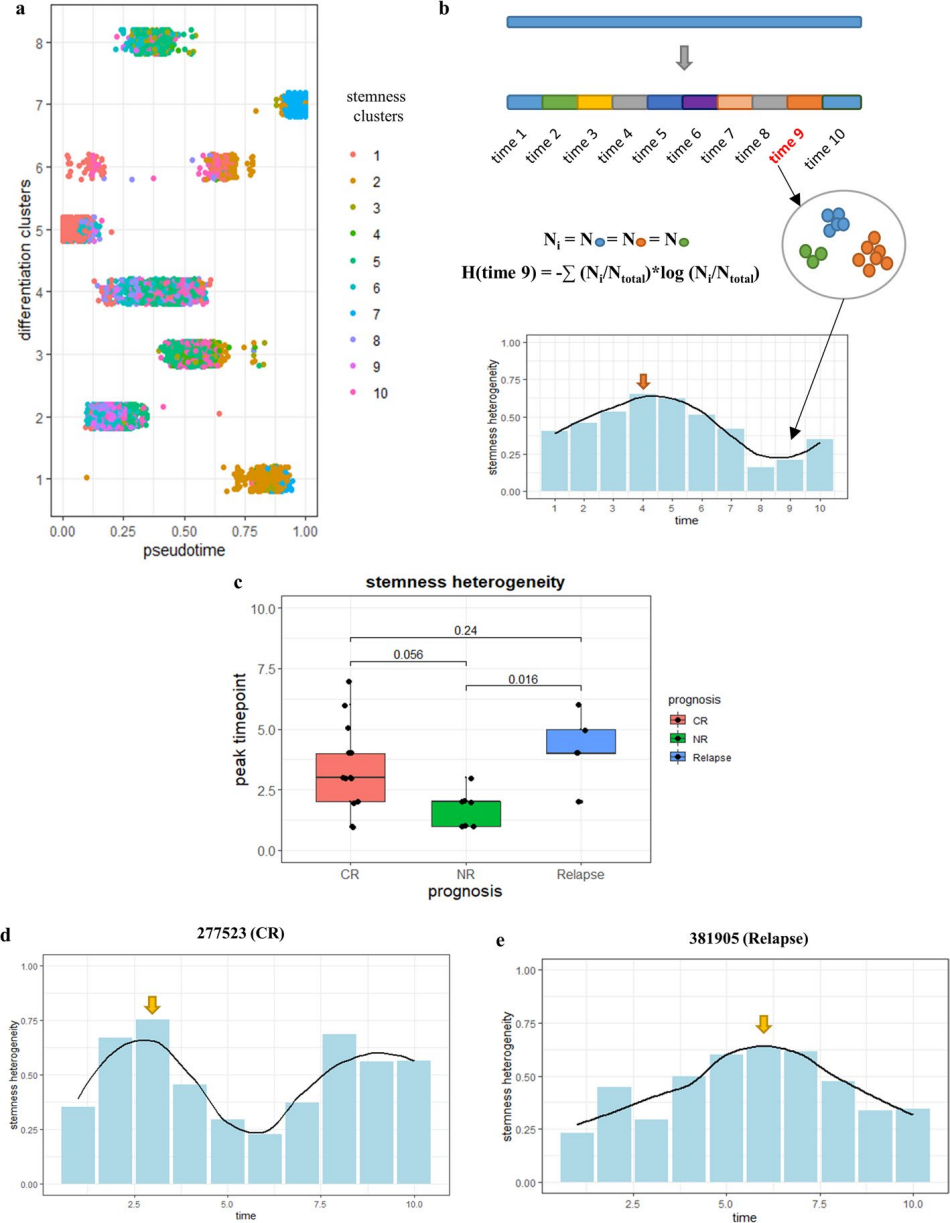
Surface stemness antigen expression heterogeneity peak point distribution on the differentiation trajectory. **a** Stemness clusters derived from 15-antigen stemness panel distribute on pseudotime axis; **b** stemness heterogeneity temporal distribution diagram. Cells with stemness clusters and counts in each time period were extracted, and stemness heterogeneity was calculated based on shannon entropy [H(x)]. Then the stemness heterogeneity of every time period was ordered along pseudotime axis with wavy shape; **c** stemness heterogeneity peak time counted in different prognosis type of samples; **d**, **e** stemness heterogeneity temporal distribution of complete-remission (CR) (no. 277523) and relapse (no. 381905). The peak timepoint was labelled by yellow arrow

Shannon entropy is a concept that is used to generally evaluate system complexity, including the quantification of morphological and molecular diversity in AML via flow cytometry [26], and has also been used to predict prognosis from single-cell RNA-sequencing data [27]. To investigate stemness by surface antigen profiling, we selected 15 stem-related surface antigens from a previous publication [28] (Additional File 1: Table S2) and proposed a ‘stemness heterogeneity’ index for the measurement of expression complexity of the 15 antigens (Fig. 6a). Subsequently, the blasts were divided into 10 parts according to the pseudotime values of single cells. The stemness heterogeneity index of each time period was calculated and plotted on the differentiation axis (Fig. 6b). According to the distribution of every sample, samples with CR (n = 14) and NR (n = 7) presented an earlier mean peak time (Fig. 6c). The first peak of heterogeneity appeared earlier in the CR group (Fig. 6d, 9/13 samples with the peak early than time point 3) than in the relapse group (Fig. 6e 4/5 samples with the peak after time point 3) (*p* = 0.24), suggesting that the time peak could serve as an indicator of relapse at the proteomic level. In addition, there was an earlier trend of peak time point of stemness heterogeneity in the NR samples than in the CR samples (*p* = 0.056), which might be related to the proliferation dynamics of blood system that would be discussed later, but makes NR group hardly to be distinguished with CR. Together, these results indicated the potential for the stemness heterogeneity temporal distribution to be used for diagnostic prediction. The dynamics of stemness heterogeneity for each sample is shown Additional File 1: Table S4 and Fig. S6 shows the stem-entropy dynamics of the five relapse samples.

### Hierarchical stemness heterogeneity and 17-gene stemness score at the single-cell RNA level

**Fig. 7:**
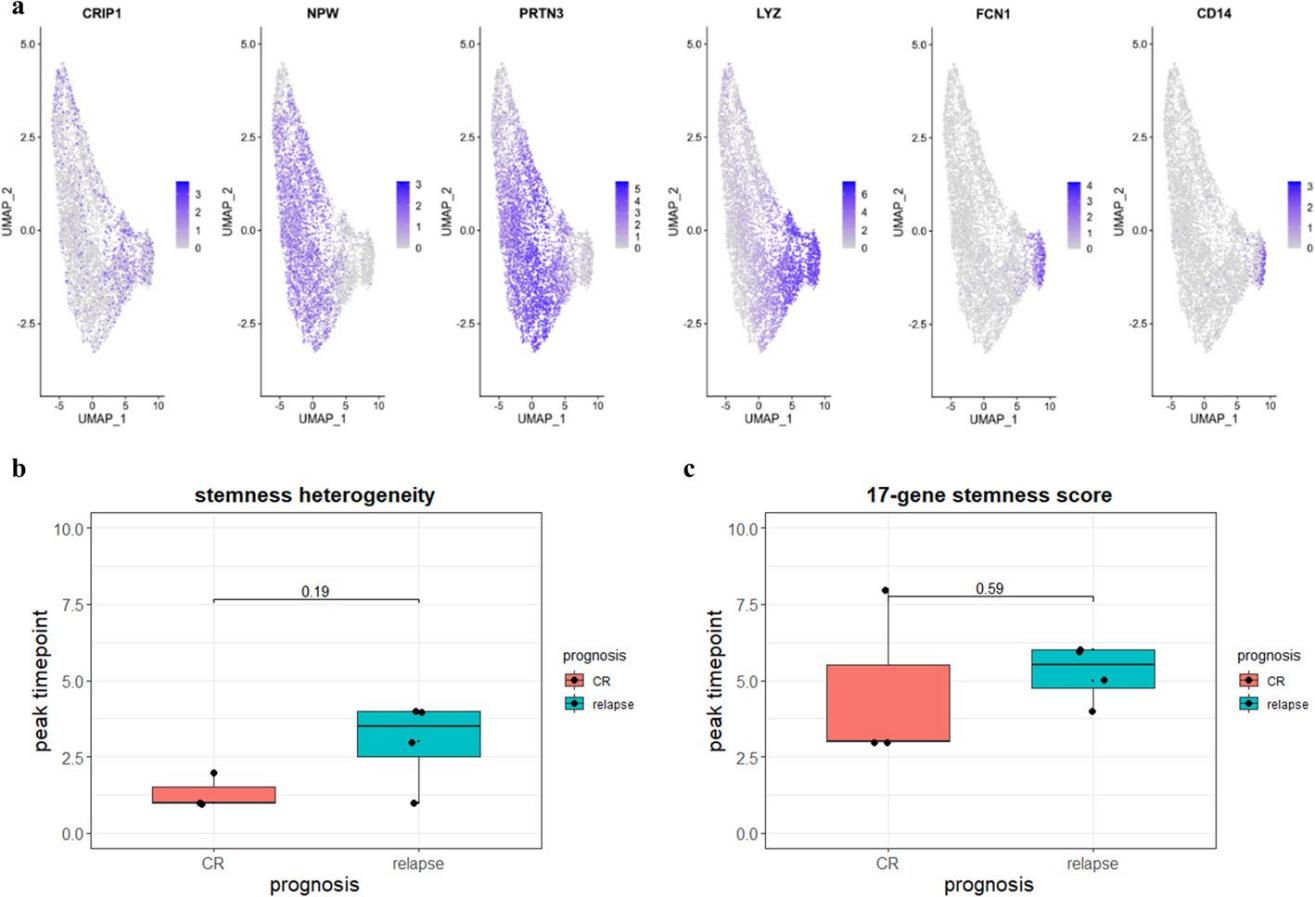
Stemness heterogeneity distribution was tested at single-cell RNA level. **a** Reported specific biomarkers expressed hierarchically in the blast. Respectively, mature myeloid blasts always express FCN1 and CD14, monocytes express LYZ, abnormal monocytes in ealier stage express PRTN3 and located in intermediate stage, while CRIP1 and NPW express in CD34^+^/CD117^+^ clones are regarded as primitive biomarker as published results; **b** stemness heterogeneity peak time point distribution in pseudotime. The stemness-related RNA molecules referred to the genes in 17-gene stemness score system; **c** 17-gene stemness score trough point distribution in pseudotime

Finally, we tested the proteome-based stemness heterogeneity dynamics using scRNA-seq of three CR patients and four relapse patients. After pre-processing and cell filtering, the remaining monocytes aligned on the trajectory depended on differentiation-related RNA molecules [15]. Specific markers representing the differentiation stages of AML were matched to the data reported in the original article, including mature molecules such as FCN1 and CD14 and early markers such as CRIP1 and NPW (Fig. 7a) [14]. The datasets were integrated to ensure a unified UMAP space distribution and trajectory. After inference with the SCORPIUS algorithm, the trajectory of each sample was drawn and a pseudotime value was assigned for each cell as an indicator of the degree of differentiation, similar to the analysis workflow of mass cytometry.

Stem-related RNA molecules were obtained from the molecules detected in the 17-gene stemness scoring system; the 17 core transcriptional molecules were significantly expressed in LSCs and showed potential for predicting a poor outcome. Stemness heterogeneity depends on the 17 stem-related molecules that were calculated in a manner similar to the mass cytometry. By comparing the time point of the initial stemness heterogeneity peak, we identified the late appearance of the first peak in most of the relapse samples (3/4, *p* = 0.19); the peak time point occurred after time 3, in contrast to the first peak point at time 1 or 2 in the CR samples (Fig. 7b). Considering the correlation between gene transcription and protein production, it follows that the distribution of stemness heterogeneity at the single-cell RNA level could confirm the results arising from mass cytometry, thus predicting a poor prognosis via the late appearance of the first stemness heterogeneity peak.

The progression of AML is accompanied by an overall down-regulation of founding genes and formation of mature phenotypes. During this process, changes of stemness along the trajectory implied an alternation between earlier and later molecules. Therefore, we assumed that the first peak of stemness heterogeneity was attributed to the initial molecules and that stemness would gradually reduce over time due to competition with evolution-related molecules. We used the 17-gene stemness score as a benchmark to evaluate the risk for AML relapse. The pseudotime point of the first trough in the 17-gene stemness score was defined as the remaining AML-founding factor capability. We found that four relapse patients approached the time point of this first trough later than the three patients who achieved CR; these patients showed relatively consistent stemness heterogeneity (Fig. 7c). However, the difference in the peak time point for the 17-gene stemness score was less significant (*p* = 0.59) than that of the 17-gene stemness heterogeneity (*p* = 0.19), which might indicate a better utility of a Shannon entropy-based stemness molecular complexity index.

## Discussion

Significant heterogeneity builds up during AML progression, thus generating blasts with temporal information of drug resistance, relapse, and phenotype transformation. To explore these dynamics of heterogeneity with regard to specific timescales, we chose mass cytometry to profile the surface antigen landscape of single myelocytes. As a multi-protein single-cell detection technique, mass cytometry provides a similar but more elaborate form of analysis than conventional flow cytometry, thus creating greater alignment between results and clinical indices. Using the surface antigen landscape, we were able to capture similarities and differences among cells, which were then used in the screening of temporal features among clinical samples for AML prognosis prediction.

In this study, we abandoned the conventional strategy of considering healthy myeloid clones as granular “landmarks” and developed a new method for continuous time-series analysis in AML research. The new workflow proposed herein yields a differentiation pseudotime for not only each cell but also every tested molecule. On the continuous differentiation trajectory, we were able to narrow down the primitive clones by selecting the initial period, and in analysing drug efficacy, we were able to clarify signal responses much more precisely at certain time points: at the initial, very early, mid-to-late, and end stages. In addition, we found that a small number of surface antigens (12 identified from the dataset used in the present study) is sufficient to allocate a correct pseudotime to each cell. This approach provides the capability of optimizing detection channels for other aspects of medical research, such as signal molecule drug responses and the evaluation of prognosis based on stemness.

In contrast to earlier analyses, which investigated more branches or nodes and thus led to a more complex differentiation trajectory, we adopted a single-linear trajectory inference algorithm to simplify the pattern of AML differentiation. First, we selected 12 antigens by DANCE to identify the commonality of differentiation patterns among patients and then investigated the intra- and inter-tumour heterogeneity along the trajectory. Although we were able to incorporate the temporal behaviour of molecules as a similar temporal ‘yardstick’ in this study, the commonality of different trajectories needs further improvement to apply this method to a greater number of patients. Here, we conceived a group of artificial references for which biomarkers related to standard differentiation were added to specimens before mass cytometry detection. These references are expected to align more efficiently with cells on a uniform trajectory. In addition, the pipeline for analysis and algorithms require certain modifications if they are to automatically recognize the correct global and local direction of a trajectory.

The trajectory analysis workflow provides us new perspective of AML heterogeneity, and the temporal pattern might indicate prognosis evaluation potential. CD11c early expression is an indicator of good prognosis, but the research is restricted by the sample size. To find out the mechanism of CD11c during AML development, we assumed that CD11c was normally expressed on the surface of mature blasts. As an adhesion molecule, it plays a role in cell-to-cell interaction and recognition, which might influence the chemotherapy prognosis of AML cells. To test the first hypothesis, we investigated the CD11c-CD18 interaction in the STRING website (https://cn.string-db.org/) (co-expression score: 0.998). According to the literature, the early expression of CD11c and CD18 form integrin for the binding of C3b complement [25], thus indicating an immune enhancement to the AML blast, especially primitive clones (Figure S5).

The prognosis evaluation factors identified herein not only act alone in a single molecule but also act in combination. For example, to consider the plastic phenotype related to AML relapse, we regarded relapse-related stemness as a feature of each cell and proposed a stemness heterogeneity index based on Shannon entropy to reflect the expression complexity of 15 stemness-related molecules. This indicator fully considers the plasticity of stemness-related surface molecules, which are expressed without regularity and modality and with no standard landscape in different patients. Although we observed trends of change between samples from patients with CR and relapse outcomes, the difference of stemness heterogeneity at the protein level was not significant as expected. Therefore, this prognosis evaluation index needs to be further optimized for the prediction of poor outcomes in a more efficient and precise manner.

The 17-gene stemness score provides specific parameters relating to stemness heterogeneity at the scRNA level. This analysis also suggested that RNA, inner signal proteins, or other molecules could be useful stemness-related factors for relapse prediction, which might require the use of single-cell multi-omics platforms for further detection. Future research relating to stemness heterogeneity should be expanded beyond the tested molecules to include not only surface antigens but also intracellular signals or transcriptional factors. Moreover, it might be valuable to considering weighting the importance of each molecule to increase the accuracy of the model. In addition, the scRNA-seq datasets derived from adult patients produced the same trend for mass cytometry-deduced stemness heterogeneity as found for the paediatric specimens, thus suggesting that this index might be suitable for all AML samples. Owing to the limited number of samples, we could not investigate more temporal features associated with classified phenotypes and genotypes. However, we have provided analytical strategies that focus on commonalities of the blood system in each patient without considering the disease sub-type or molecular abnormalities. Furthermore, our definition of stemness heterogeneity measures the stemness of each cell. Consequently, it can be assumed that the degree of stemness between healthy and AML cells at the same stage would differ; that is, stemness is lower in healthier states than in disease states. If our hypothesis is valid, then the stemness heterogeneity index will eliminate the influence of healthy myeloid cells that are resident in AML blasts.

Additionally, we speculated the earliest peak times of stemness heterogeneity distribution in NR samples are associated with their proliferation rate, which might be influenced by various genomic alterations and heterogeneity of initial clones. The beginning sub-clones interact with each other to survive. In the relapse group, few resistant cells need to remain dormant among other proliferating populations before being selected by chemotherapy drugs, while the non-remission samples have a more stable blood system with lower competition. Therefore, the NR blood systems might have a lower differentiation rate. The apoptosis rate is higher in CR group than NR in reported result [28], which could also indirectly explain the different peak time between these two patients. The future work could closely test the speculation about competition and proliferation rate with the different molecular platforms.

In summary, further research should focus on optimization of the analysis pipeline, including consistency improvement, precise cell-stage determination, and the expansion of test molecules to other detection platforms, or even multi-omics research. In future clinical research, it will be important to test a greater number of AML specimens (both paediatric and adult) to identify indicators to guide the therapeutic strategy in the early stages. Furthermore, since differentiation induction therapy has shown good efficacy for AML treatment (ATRA to M3 AML patients) [29], this new form of temporal analysis exhibits good potential as a tool for differentiation induction, drug response monitoring, and the assessment of efficacy.

## Conclusion

In this study, we developed a novel trajectory analysis pipeline that recreates the phenotypic dynamics of AML progression based on a single-cell surface antigen landscape. We then optimized the model by determining a common evolution pattern among patients through the proposed DANCE feature selection strategy. The continuous presentation of AML progression reflects an actual differentiation order of single cells and their expressed molecules. On the hierarchy, we identified that abnormal CD11c expression at the primitive stage of AML differentiation predicts a good chemotherapy outcome. We further proposed stemness heterogeneity as an indicator of relapse risk, suggesting that detection of the initial stemness maintenance might contribute to a more accurate prognosis evaluation. Overall, our proposed temporal analysis strategy provides insight into the heterogeneity of AML and may serve as a reference for clinical diagnosis and prognosis.

### Supplementary Information

**Additional file 1.** Sample information, antibody labelling, intermediate data and supplementary figures.

## Data Availability

The mass cytometry AML datasets and code generated during the current study are available in the Mendeley Data repository (https://data.mendeley.com/datasets/56zh5g7bfw/4). scRNA-seq data was downloaded from the National Omics Data Encyclopedia (NODE) (http://www.biosino.org/node/project/detail/OEP000629) with the permission of author [14]. The follow-up information of patients and the relevant data are presented in supplementary materials. Accessing more details need to contact co-responding authors.

## Abbreviations

AML: Acute myeloid leukaemia
CR: Complete response
CyTOF: Cytometry time-of-flight
DANCE: Dispersive antigens in neighbouring clusters exhibition
NR: Non-remission
LOESS: Locally weighted regression
LSC: Leukaemia stem cell
