# Supplementary material for: Detection of continuous hierarchical heterogeneity by single-cell surface antigen analysis in the prognosis evaluation of acute myeloid leukaemia
Source: BMC Bioinformatics. 2023 Nov 28;24:450. doi: 10.1186/s12859-023-05561-0 (PMC10683216; doi:10.1186/s12859-023-05561-0)
Supplement: Supplementary file 1 — Additional file 1. Sample information, antibody labelling, intermediate data and supplementary figures. [file 12859_2023_5561_MOESM1_ESM.docx]

**Table S1. Detail information of 43 paediatric AML clinical samples**

| **sample ID** | **subtype** | **prognosis** | **genetic abnormalities** | **treatment** |
| --- | --- | --- | --- | --- |
| 287167 | M0 | NR | MLL rearrangement | chemotherapy |
| 280377 | M2 | CR | NPM1 mutat | chemotherapy+transplantation |
| 306443 | M2 | loss to follow-up | FLT3-ITD positive | chemotherapy |
| 381905 | M2 | relapse and death | - | chemotherapy |
| 562778 | M2 | CR | Double CEBPA mutations | chemotherapy |
| 363858 | M2 | CR | Double CEBPA mutations | chemotherapy |
| 570407 | M2a | CR | Double CEBPA mutations; CSF3R, RAD21, GATA2, RBBP6, BRAF, SH2B3 mutant | chemotherapy |
| 251995 | M2a | death outside hospital | - | induction chemotherapy |
| 255667 | M2a | NR and death | - | chemotherapy |
| 296083 | M2b | relapse and death | AML1-ETO+ | chemotherapy |
| 568283 | M2b | CR | AML1-ETO+ | chemotherapy+dasatinib |
| 239077 | M2b | CR | AML1-ETO+ | chemotherapy |
| 270748 | M2b | CR | AML1-ETO+ | chemotherapy |
| 274360 | M2b | CR | AML1-ETO+ | chemotherapy |
| 277523 | M2b | CR | AML1-ETO+; C-KIT mutant | chemotherapy |
| 326944 | M2b | CR | AML1-ETO+ | chemotherapy |
| 311782 | M2b | die of infection | AML1-ETO+ | chemotherapy |
| 571310 | M4 | relapse | NPM1, GATA2, KIT, BCORL1 mutant | chemotherapy |
| 234628 | M4 | relapse and death | - | chemotherapy |
| 288221 | M4b | dead after transplantation | CBFβ-MYH11+ | chemotherapy+transplantation |
| 557507 | M4b | loss to follow-up | IL7R, KRAS, PTPN11, FLT3, EP300 mutant | chemotherapy |
| 365663 | M4EO | loss to follow-up | CBFB/MYH11+ | chemotherapy |
| 577751 | M4eo | CR | CBFβ-MYH11+; NRAS mutant; KIT mutant; DDX41 mutant; NPM1 mutant | chemotherapy+dasatinib |
| 268078 | M4Eo | NR after therapy, loss to follow-up | CBFB/MYH11+ | chemotherapy |
| 236829 | M4Eo | CR | CBFB/MYH11+ | chemotherapy |
| 237312 | M4Eo | CR | CBFB/MYH11+ | chemotherapy |
| 567143 | M5 | CR | KRAS mutant; XPO1 mutant | chemotherapy |
| 569255 | M5 | CR | RUNX1 mutant | chemotherapy |
| 233501 | M5 | relapse and death | - | chemotherapy |
| 385786 | M5 | CR | MLL-AF9 + | chemotherapy |
| 311598 | M5 | relapse and death | - | chemotherapy |
| 346095 | M5 | CR | FLT3-ITD positive | chemotherapy |
| 326960 | M5 | NR | FLT3-ITD positive | chemotherapy |
| 566912 | M5 | CR | FLT3-ITD positive | chemotherapy |
| 288903 | M5 | PR and death | MLL rearrangement | chemotherapy |
| 257511 | M5 | CR | - | chemotherapy+transplantation |
| **sample ID** | **subtype** | **prognosis** | **genetic abnormalities** | **treatment** |
| 263038 | M5 | NR and death | - | chemotherapy |
| 309910 | M5 | die of infection | MLL rearrangement | induction chemotherapy |
| 586682 | M5 | CR | MLL-AF9+; FLT3 mutant | chemotherapy |
| 238395 | M5 | NR and death | - | chemotherapy |
| 566765 | M5+MDS | NR | KRAS mutant; FBXW7 mutant | chemotherapy |
| 240164 | M6 | die of cerebral hemorrhage | - | induction chemotherapy |
| 233770 | M7 | CR | - | chemotherapy |

Abbreviations: CR, complete remission; NR, non-remission; PR, partial remission.

**Table S2. Antibody-lanthanide conjunction and application of the antibodies**

| **Antibodies**  **(cat no.)** | **company** | **Labelling lanthanides (Fluidigm Inc., part no.)** | **functions** |
| --- | --- | --- | --- |
| CD45 (#304045) | BioLegend | Pr141 (#S00014) | differentiation |
| CD47 (#323102) | BioLegend | Nd142 (#S00015) | stemness |
| CD117 (#313223) | BioLegend | Nd143 (#S00016) | differentiation & stemness |
| CD38 (#303535) | BioLegend | Sm144 (#S00017) | differentiation & stemness |
| CD163 (#326502) | BioLegend | Nd145 (#S00018) | AML diagnosis |
| CD64 (#305029) | BioLegend | Nd146 (#S00019) | differentiation |
| CD11c (#301639) | BioLegend | Nd147 (#S00020) | differentiation |
| CD16 (#302051) | BioLegend | Nd148 (#S00021) | differentiation |
| CD34 (#343531) | BioLegend | Sm149 (#S00022) | differentiation & stemness |
| CD43 (#343202) | BioLegend | Nd150 (#S00023) | diagnosis |
| CD123 (#306027) | BioLegend | Eu151 (#S00024) | stemness |
| CD13 (#301717) | BioLegend | Sm152 (#S00025) | differentiation & stemness |
| CD303 (#354215) | BioLegend | Eu153 (#S00026) | diagnosis |
| CD3 (#300443) | BioLegend | Sm154 (#S00027) | T lymphocyte biomarker |
| CD24 (#311127) | BioLegend | Gd155 (#S00098) | differentiation |
| CD36 (#336215) | BioLegend | Gd156 (#S00028) | differentiation |
| CD44 (#338811) | BioLegend | Gd158 (#S00029) | differentiation & stemness |
| Tim3 (#345019) | BioLegend | Tb159 (#S00030) | stemness |
| CD235a (#306615) | BioLegend | Gd160 (#S00031) | diagnosis |
| CD244 (#393502) | BioLegend | Dy161 (#S00102) | stemness |
| CD18 (#373402) | BioLegend | Dy162 (#S00032) | differentiation & stemness |
| CD35 (#332402) | BioLegend | Dy163 (#S00104) | differentiation |
| CD19 (#396302) | BioLegend | Ho165 (#S00034) | B lymphocyte biomarker |
| CD7 (#343111) | BioLegend | Er166 (#S00035) | stemness |
| CD11b (#301337) | BioLegend | Er167 (#S00036) | differentiation |
| CD55 (#311302) | BioLegend | Er168 (#S00037) | differentiation & stemness |
| CD33 (#303419) | BioLegend | Tm169 (#S00038) | differentiation & stemness |
| CD45RA (#304143) | BioLegend | Er170 (#S00039) | diagnosis |
| CD96 (#338402) | BioLegend | Yb171 (#S00040) | stemness |
| CD15 (#323035) | BioLegend | Yb172 (#S00041) | differentiation |
| HLA-DR (#307651) | BioLegend | Yb173 (#S00106) | differentiation & stemness |
| CD4 (#300541) | BioLegend | Yb174 (#S00042) | differentiation |
| CD14 (#301843) | BioLegend | Lu175 (#S00043) | differentiation |
| CD56 (#318345) | BioLegend | Yb176 (#S00044) | NK cell biomarker |
| CD61 | Fluidigm | Bi209 (#3209001B) | diagnosis |
| CD41 | Fluidigm | Y89 (#3089004B) | diagnosis |

**Table S3. Selected features after performing “dispersive antigens in neighbouring clusters exhibition (DANCE)” in each sample**

| **subtype** | **Sample ID** | **Selected features** |
| --- | --- | --- |
| **M0** | 287167 | CD45, CD38, CD24, CD44, CD18, CD55, CD33, CD15, HLA-DR, CD4, CD11b, CD34 |
| **M2** | 280377 | CD45, CD38, CD11c, CD36, CD44, CD18, CD35, CD11b, CD33, CD15, HLA-DR, CD4, CD14, CD34 |
|  | 306443 | CD45, CD117, CD38, CD64, CD34, CD13, CD44, CD18, CD33, HLA-DR, CD11b |
|  | 381905 | CD45, CD117, CD38, CD64, CD34, CD13, CD36, CD44, CD35, CD11b, CD55, CD33, HLA-DR, CD4, CD14 |
|  | 562778 | CD45, CD117, CD38, CD64, CD34, CD44, CD18, CD55, CD33, HLA-DR, CD4, CD11b |
|  | 363858 | CD45, CD38, CD64, CD34, CD13, CD36, CD44, CD18, CD35, CD55, CD33, CD4, CD11b |
| **M2a** | 570407 | CD45, CD117, CD38, CD34, CD13, CD36, CD44, CD18, CD11b, CD33, HLA-DR, CD4, CD14 |
|  | 251995 | CD45, CD117, CD38, CD64, CD34, CD13, CD36, CD44, CD18, CD35, CD11b, CD55, CD33, HLA-DR, CD4, CD14 |
|  | 255677 | CD45, CD117, CD38, CD64, CD34, CD44, CD18, CD11b, CD55, CD33, HLA-DR, CD4 |
| **M2b** | 296083 | CD45, CD117, CD38, CD34, CD13, CD36, CD44, CD18, CD35, CD55, CD33, CD15, CD4, CD11b |
|  | 568283 | CD45, CD117, CD38, CD64, CD34, CD13, CD36, CD44, CD18, CD35, CD33, CD15, CD4, CD11b |
|  | 239077 | CD45, CD117, CD38, CD64, CD11c, CD16, CD34, CD13, CD36, CD44, CD18, CD35, CD11b, CD33, CD15, HLA-DR, CD4 |
|  | 270748 | CD45, CD117, CD38, CD64, CD34, CD13, CD44, CD18, CD55, CD33, CD15, CD4, CD11b |
|  | 274360 | CD45, CD117, CD38, CD34, CD13, CD44, CD18, CD11b, CD33, CD15, CD4, CD14 |
|  | 277523 | CD45 , CD117 , CD38 , CD64 , CD34 , CD13 , CD44 , CD18 , CD11b , CD33 , CD15 , HLA-DR , CD4 |
|  | 326944 | CD45, CD117, CD38, CD64, CD11c, CD34, CD36, CD44, CD18, CD11b, CD55, CD33, CD15, HLA-DR, CD4 |
|  | 311782 | CD45, CD117, CD38, CD64, CD16, CD34, CD13, CD44, CD18, CD35, CD11b, CD33, CD15, HLA-DR |
| **M4** | 571310 | CD45, CD117, CD38, CD64, CD34, CD13, CD36, CD44, CD18, CD35, CD55, CD33, HLA-DR, CD4, CD14, CD11b |
|  | 234628 | CD45, CD117, CD38, CD16, CD34, CD18, CD35, CD11b, CD33, CD15, CD14 |
| **M4b** | 288221 | CD45, CD117, CD38, CD11c, CD34, CD13, CD44, CD35, CD11b , CD33, HLA-DR, CD4 |
|  | 557507 | CD45, CD117, CD38, CD34, CD13, CD36, CD44, CD18, CD11b, CD55, CD33, HLA-DR, CD4 |
| **M4eo** | 365663 | CD45, CD117, CD38, CD64, CD34, CD13, CD36, CD44, CD18, CD35, CD11b, CD55, CD33, CD15, CD14 |
|  | 577751 | CD45, CD117, CD38, CD64, CD11c, CD34, CD13, CD44, CD18, CD35, CD11b, CD55, CD33, HLA-DR, CD4, CD14 |
|  | 268078 | CD45, CD117, CD38, CD64, CD11c, CD34, CD13, CD36, CD44, CD18, CD11b, CD55, CD33, CD15, HLA-DR, CD4, CD14 |
|  | 236829 | CD45, CD117, CD38, CD34, CD13, CD36, CD44, CD18, CD35, CD11b, CD55, CD33, CD15, HLA-DR, CD4, CD14 |
|  | 237312 | CD45, CD38, CD64, CD16, CD34, CD13, CD36, CD44, CD18, CD11b, CD55, CD33 |
| **M5** | 567143 | CD45, CD38, CD64, CD34, CD36, CD44, CD18, CD35, CD55, CD33, CD15, HLA-DR, CD4, CD11b |
|  | 569255 | CD45 , CD117 , CD38 , CD34 , CD13 , CD36 , CD44 , CD18 , CD55 , CD33 , CD15 , HLA-DR , CD4 , CD11b |
|  | 233501 | CD45, CD38, CD64, CD11c, CD34, CD13, CD44, CD18, CD11b, CD55, CD33, HLA-DR, CD4, CD14 |
|  | 385786 | CD45, CD38, CD64, CD36, CD44, CD18, CD35, CD11b, CD55, CD33, HLA-DR, CD4, CD14, CD34 |
|  | 311598 | CD45, CD117, CD38, CD64, CD34, CD13, CD36, CD44, CD18, CD11b, CD55, CD33, HLA-DR, CD4 |
|  | 566765 | CD45, CD117, CD38, CD34, CD44, CD18, CD35, CD11b, CD33, CD4, CD14 |
|  | 346095 | CD45, CD38, CD64, CD34, CD13, CD36, CD44, CD55, CD33, CD4, CD11b |
|  | 326960 | CD45 , CD117 , CD38 , CD64 , CD11c , CD34 , CD13 , CD36 , CD44 , CD11b , CD55 , CD33 |
|  | 566912 | CD45, CD38, CD34, CD13, CD36, CD44, CD18, CD33, HLA-DR, CD4, CD11b |
|  | 228903 | CD45, CD38, CD64, CD34, CD13, CD44, CD55, CD33, CD4, CD11b |
|  | 257511 | CD45, CD117, CD38, CD64, CD34, CD13, CD36, CD44, CD18, CD11b, CD55, CD33, HLA-DR, CD4 |
|  | 263038 | CD45 , CD38 , CD64 , CD11c , CD34 , CD13 , CD36 , CD44 , CD18 , CD35 , CD11b , CD55 , CD33 , CD14 |
|  | 309910 | CD45, CD38, CD64, CD11c, CD13, CD36, CD44, CD18, CD55, CD33, CD15, HLA-DR, CD4, CD14, CD11b, CD34 |
|  | 586682 | CD45, CD117, CD38, CD64, CD11c, CD36, CD44, CD18, CD55, CD33, CD15, HLA-DR, CD4, CD14, CD11b, CD34 |
| **subtype** | **Sample ID** | **Selected features** |
|  | 238395 | CD45 , CD38 , CD64 , CD11c , CD34 , CD13 , CD36 , CD44 , CD11b , CD55 , CD33 , HLA-DR , CD4 |
| **M6** | 240164 | CD45, CD117, CD38, CD64, CD11c, CD34, CD13, CD36, CD44, CD18, CD35, CD11b, CD55, CD33, HLA-DR, CD4 |
| **M7** | 233770 | CD45, CD38, CD36, CD44, CD35, CD55, CD33, CD4, CD11b, CD34 |

**Table S4. The time sequencing dynamics of stemness heterogeneity peak timepoint in 26 samples**

| **sample ID** | **prognosis** | **time1** | **time2** | **time3** | **time4** | **time5** | **time6** | **time7** | **time8** | **time9** | **time10** |
| --- | --- | --- | --- | --- | --- | --- | --- | --- | --- | --- | --- |
| **287167** | NR | 0.6937 | 0.8870 | 0.8319 | 0.7697 | 0.8960 | 0.8016 | 0.0049 | 0.0000 | 0.0000 | 0.2117 |
| **280377** | CR | 0.2117 | 0.5208 | 0.5724 | 0.4413 | 0.4496 | 0.4558 | 0.5010 | 0.5881 | 0.5142 | 0.4481 |
| **381905** | Relapse | 0.2603 | 0.3880 | 0.2604 | 0.5533 | 0.5304 | 0.5692 | 0.5963 | 0.4684 | 0.4511 | 0.3385 |
| **562778** | CR | 0.5238 | 0.7204 | 0.6915 | 0.5481 | 0.5204 | 0.7449 | 0.7405 | 0.4914 | 0.3323 | 0.3516 |
| **363858** | CR | 0.2713 | 0.4772 | 0.7310 | 0.6329 | 0.6704 | 0.7600 | 0.6654 | 0.4515 | 0.3178 | 0.3692 |
| **570407** | CR | 0.2888 | 0.6823 | 0.6529 | 0.6546 | 0.6853 | 0.6232 | 0.5267 | 0.2892 | 0.3275 | 0.2887 |
| **255667** | NR | 0.2877 | 0.6502 | 0.5481 | 0.5181 | 0.5742 | 0.6425 | 0.6694 | 0.3923 | 0.2364 | 0.2096 |
| **296083** | Relapse | 0.3288 | 0.3570 | 0.4021 | 0.5701 | 0.5703 | 0.4380 | 0.5040 | 0.4805 | 0.5107 | 0.6067 |
| **568283** | CR | 0.5664 | 0.5107 | 0.4617 | 0.3902 | 0.4917 | 0.5503 | 0.4717 | 0.2984 | 0.4537 | 0.4986 |
| **274360** | CR | 0.5437 | 0.2992 | 0.5278 | 0.2282 | 0.5923 | 0.5752 | 0.1123 | 0.5725 | 0.4232 | 0.5404 |
| **277523** | CR | 0.3528 | 0.6684 | 0.7519 | 0.4551 | 0.2938 | 0.2271 | 0.3719 | 0.6841 | 0.5599 | 0.5651 |
| **326944** | CR | 0.5836 | 0.5317 | 0.5078 | 0.5076 | 0.4900 | 0.4981 | 0.3208 | 0.3451 | 0.4856 | 0.3952 |
| **571310** | Relapse | 0.6102 | 0.4846 | 0.5146 | 0.6469 | 0.5539 | 0.3929 | 0.5102 | 0.2679 | 0.2603 | 0.3091 |
| **234628** | Relapse | 0.4547 | 0.5031 | 0.4894 | 0.4308 | 0.3894 | 0.4336 | 0.5599 | 0.4505 | 0.6284 | 0.3698 |
| **577751** | CR | 0.6319 | 0.6674 | 0.5094 | 0.3793 | 0.2940 | 0.3459 | 0.4514 | 0.5445 | 0.5489 | 0.4639 |
| **268078** | NR | 0.5108 | 0.4995 | 0.4019 | 0.4201 | 0.5643 | 0.5708 | 0.5057 | 0.5422 | 0.4959 | 0.4641 |
| **236829** | CR | 0.5044 | 0.5375 | 0.4922 | 0.6331 | 0.6378 | 0.6046 | 0.5138 | 0.5494 | 0.3213 | 0.2874 |
| **237312** | CR | 0.2373 | 0.0578 | 0.3696 | 0.4598 | 0.3941 | 0.4128 | 0.5014 | 0.6694 | 0.8079 | 0.6902 |
| **569255** | CR | 0.5466 | 0.5509 | 0.6114 | 0.5590 | 0.5826 | 0.4850 | 0.4666 | 0.4014 | 0.4354 | 0.4746 |
| **233501** | Relapse | 0.4036 | 0.3848 | 0.3478 | 0.6322 | 0.5400 | 0.5826 | 0.4711 | 0.4414 | 0.3511 | 0.5414 |
| **385786** | CR | 0.2764 | 0.4205 | 0.3277 | 0.4420 | 0.4771 | 0.5992 | 0.6902 | 0.5812 | 0.5023 | 0.4911 |
| **566765** | NR | 0.5683 | 0.8286 | 0.4545 | 0.3891 | 0.1171 | 0.3800 | 0.4252 | 0.3911 | 0.4235 | 0.3539 |
| **326960** | NR | 0.7059 | 0.3909 | 0.5451 | 0.8491 | 0.6114 | 0.1671 | 0.0000 | 0.0000 | 0.3811 | 0.3315 |
| **257511** | CR | 0.3991 | 0.3661 | 0.6257 | 0.5717 | 0.6105 | 0.5873 | 0.4585 | 0.1289 | 0.4016 | 0.5511 |
| **263038** | NR | 0.4381 | 0.3373 | 0.1728 | 0.0000 | 0.1325 | 0.4877 | 0.6426 | 0.6308 | 0.7137 | 0.8160 |
| **238395** | NR | 0.5431 | 0.6326 | 0.3825 | 0.1435 | 0.1879 | 0.4010 | 0.3544 | 0.3590 | 0.5156 | 0.6323 |

Abbreviations: CR, complete remission; NR, non-remission.

The mark lines label the peak timepoint of each sample’s stemness heterogeneity dynamics after LOESS fitting.

**Table S5. Stemness heterogeneity at single-cell RNA level dynamics on time sequencing**

|  | **sample ID** | **time1** | **time2** | **time3** | **time4** | **time5** | **time6** | **time7** | **time8** | **time9** | **time10** |
| --- | --- | --- | --- | --- | --- | --- | --- | --- | --- | --- | --- |
| **relapse** | **scAML-013P** | 0.4821 | 0.4432 | 0.5866 | 0.5871 | 0.5825 | 0.4724 | 0.3763 | 0.3694 | 0.1770 | 0.2936 |
|  | **scAML-016P** | 0.4467 | 0.5223 | 0.7482 | 0.3636 | 0.5484 | 0.6191 | 0.4663 | 0.4261 | 0.3937 | 0.1531 |
|  | **scAML-060P** | 0.3746 | 0.6088 | 0.6876 | 0.7675 | 0.5164 | 0.5747 | 0.5085 | 0.2913 | 0.3180 | 0.1858 |
|  | **scAML-070P** | 0.6975 | 0.3991 | 0.4762 | 0.3015 | 0.4543 | 0.6255 | 0.5929 | 0.5032 | 0.5082 | 0.3612 |
|  |  |  |  |  |  |  |  |  |  |  |  |
| **CR** | **scAML-072P** | 0.5894 | 0.4878 | 0.5024 | 0.5152 | 0.6830 | 0.3628 | 0.6517 | 0.5618 | 0.1957 | 0.3051 |
|  | **scAML-076P** | 0.6607 | 0.7162 | 0.4254 | 0.5696 | 0.3670 | 0.4360 | 0.5378 | 0.4956 | 0.3710 | 0.1186 |
|  | **scAML-101P** | 0.5774 | 0.6311 | 0.4555 | 0.6413 | 0.3354 | 0.3900 | 0.4273 | 0.4816 | 0.4007 | 0.4098 |

The mark lines label the peak timepoint of each sample’s stemness heterogeneity dynamics after LOESS fitting.

**Table S6. 17-gene stemness score at single-cell RNA level dynamics on time sequencing**

|  | **sample ID** | **time1** | **time2** | **time3** | **time4** | **time5** | **time6** | **time7** | **time8** | **time9** | **time10** |
| --- | --- | --- | --- | --- | --- | --- | --- | --- | --- | --- | --- |
| **relapse** | **scAML-013P** | -0.042 | -0.063 | -0.075 | -0.086 | -0.087 | -0.073 | -0.057 | -0.035 | -0.010 | -0.005 |
|  | **scAML-016P** | -0.040 | -0.091 | -0.090 | -0.102 | -0.099 | -0.101 | -0.104 | -0.086 | -0.068 | -0.037 |
|  | **scAML-060P** | -0.027 | -0.036 | -0.056 | -0.069 | -0.075 | -0.079 | -0.076 | -0.054 | -0.035 | -0.011 |
|  | **scAML-070P** | -0.065 | -0.065 | -0.077 | -0.078 | -0.083 | -0.090 | -0.084 | -0.070 | -0.058 | -0.045 |
|  |  |  |  |  |  |  |  |  |  |  |  |
| **CR** | **scAML-072P** | -0.034 | -0.038 | -0.045 | -0.035 | -0.032 | -0.050 | -0.032 | -0.055 | -0.061 | -0.060 |
|  | **scAML-076P** | -0.019 | -0.039 | -0.042 | -0.033 | -0.044 | -0.049 | -0.054 | -0.060 | -0.050 | -0.016 |
|  | **scAML-101P** | -0.034 | -0.045 | -0.037 | -0.047 | -0.060 | -0.063 | -0.067 | -0.076 | -0.065 | -0.043 |

The mark lines label the peak timepoint of each sample’s stemness heterogeneity dynamics after LOESS fitting.

**
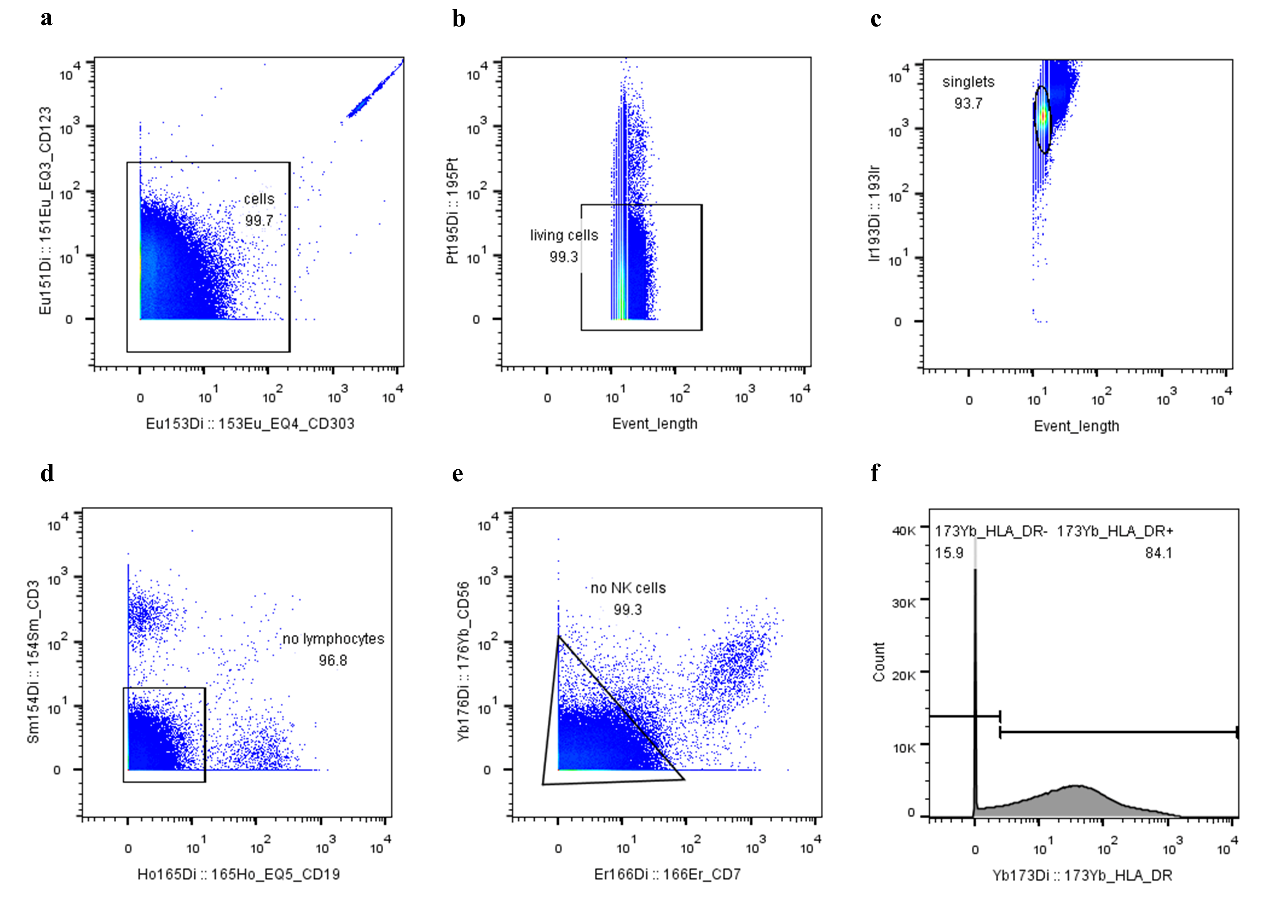
**

**Figure S1. The cell filtering strategy via flowjo.** The raw CyTOF data of 43 clinical samples was pre-

processed by filtering EQ beads (a), picking living cells with lower Pt signal (b), removing doublets with Ir-inter (c). Then the monocytes in the blast was purified through discarding T cells (CD3), B cells (CD19) (d), NK cells (CD56, e), and finally the tested AML lineage cells were reserved with HLA-DR^+^ (f)

**
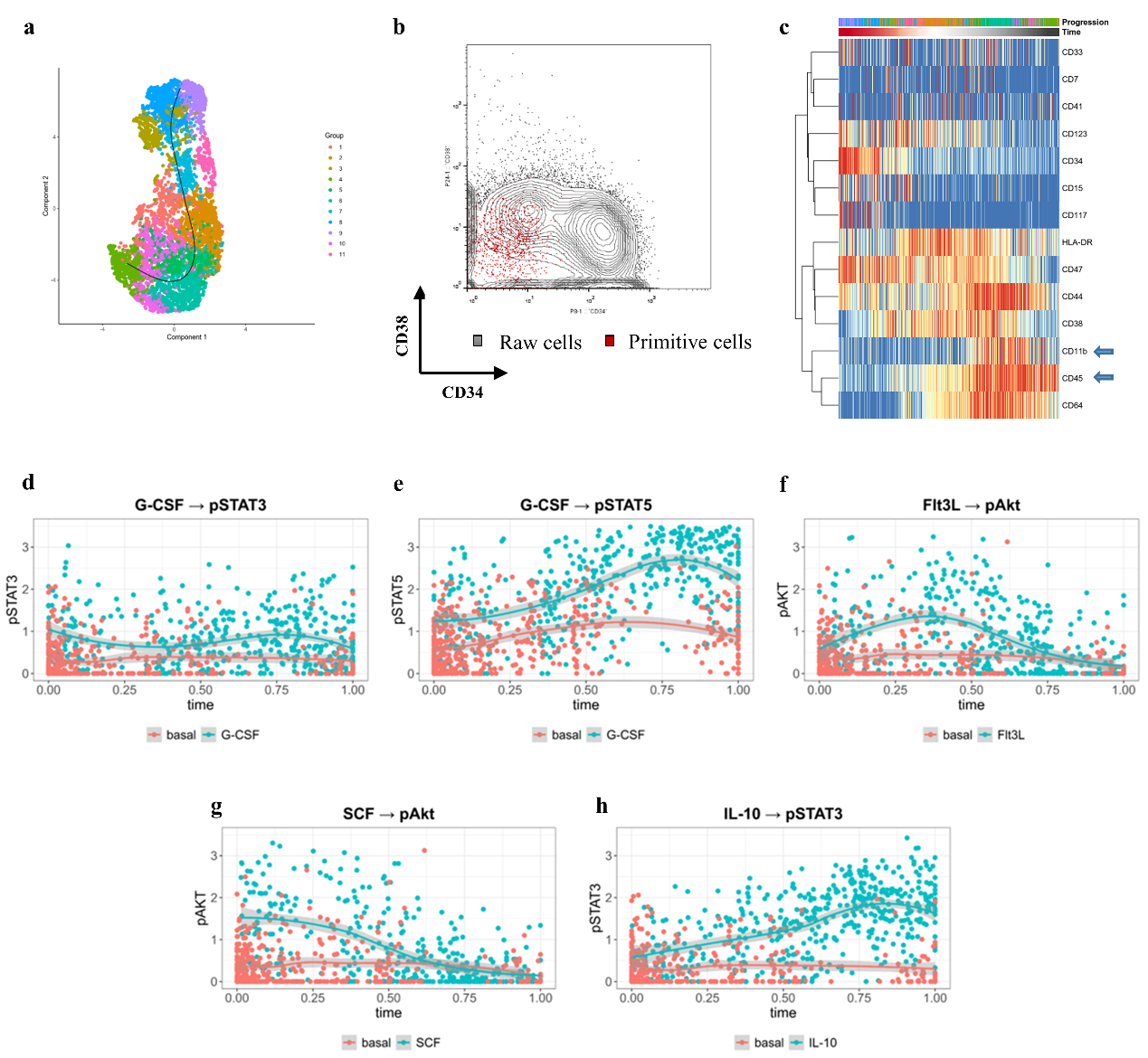
**

**Figure S2 Differentiation trajectory analysis in sample SJ05.** (a) Differentiation trajectory inference in SJ05 according to surface antigens expression; (b) Primitive cells was emphasized in red with CD34^+^ phenotype; (c) Time-series heatmap of the reported surface antigen panel in which mature blasts express CD11b^+^/CD45^high^ at the terminal; (d-h) Inner molecules temporal expression at basel (red) and drug stimulated (blue) conditions; (d) pSTAT3 achieved an overall increase during progression with the induction of G-CSF; (e) a slightly increase of pSTAT5 expression after G-CSF induction; (f and g) pAkt in earlier stage blasts upregulated with the effect of Flt3L and SCF; (h) pSTAT3 expression level was significant increased by IL-10 stimulation.

**
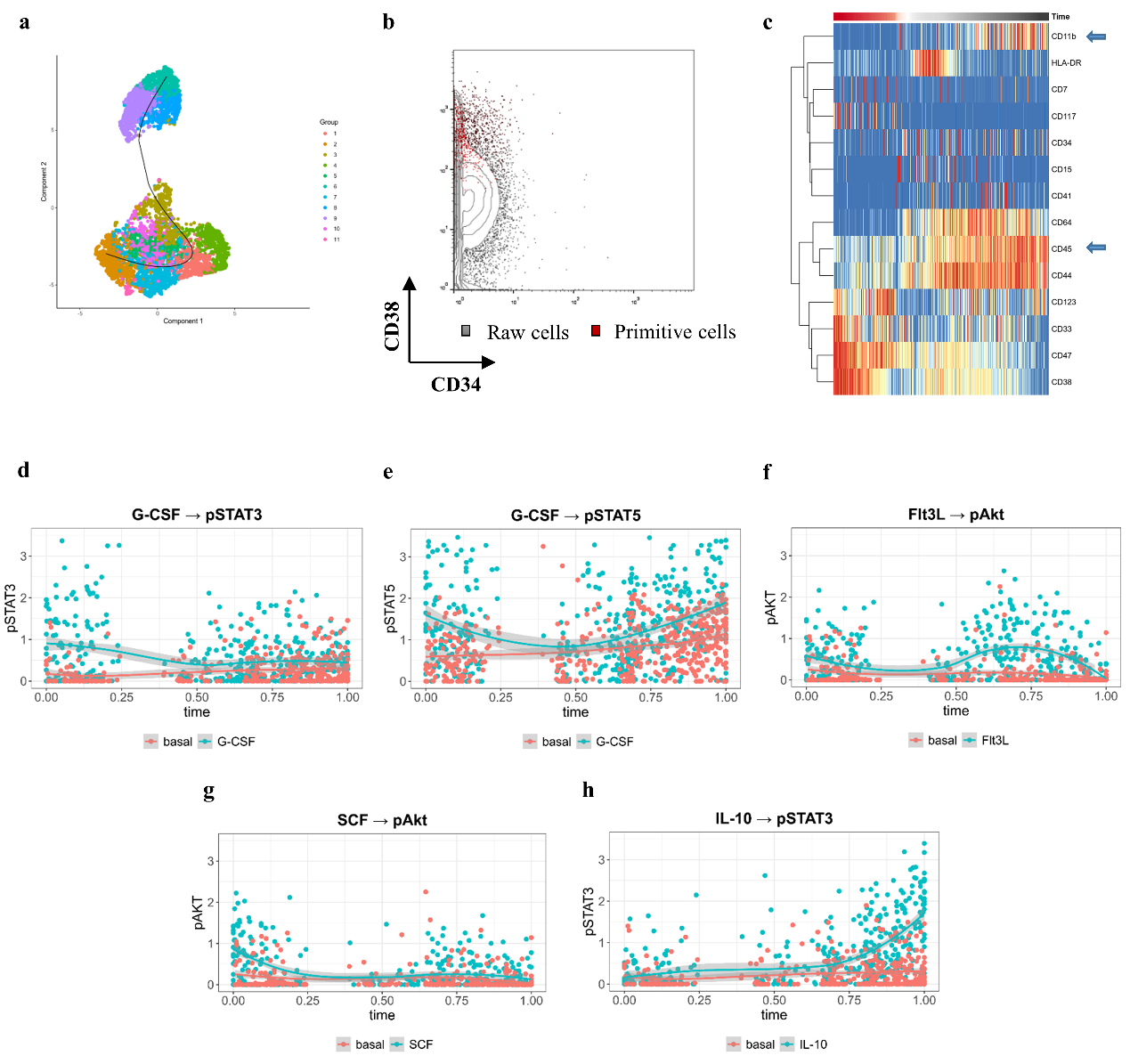
**

**Figure S3. Differentiation trajectory analysis in sample SJ16.** (a) Differentiation trajectory inference in SJ16 according to surface antigens expression; (b) Primitive cells was emphasized in red with CD34^+^ phenotype; (c) Time-series heatmap of the reported surface antigen panel in which mature blasts express CD11b^+^/CD45^high^ at the terminal; (d-h) Inner molecules temporal expression at basel (red) and drug stimulated (blue) conditions; (d) pSTAT3 increased in early stage with the induction of G-CSF; (e-g) a slightly increase of pSTAT5 expression in overall progression except terminal after drug induction; (h) pSTAT3 expression level was overall increased by IL-10 stimulation.

**
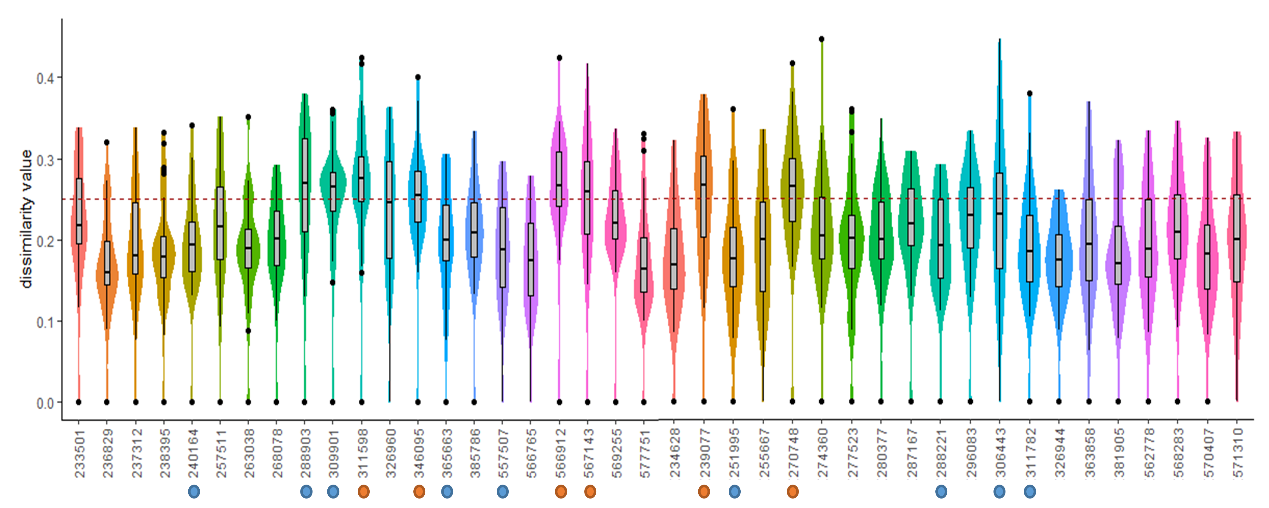
**

**Figure S4. The dissimilarity value of each sample with others presented a concentrated distribution.** Lower dissimilarity value indicated a similar differentiation pattern between two samples. In 41 samples with good trajectory fitting, nine of them including one PR (sample no. 288903) and eight with the loss of follow-up information or died of complications (blue dots) were firstly excluded in ectopic expression detection. Subsequently, six samples with lower average dissimilarity value compared to others (orange dots) would drop out of the stemness heterogeneity distribution analysis for the consistence of differentiation trajectories.


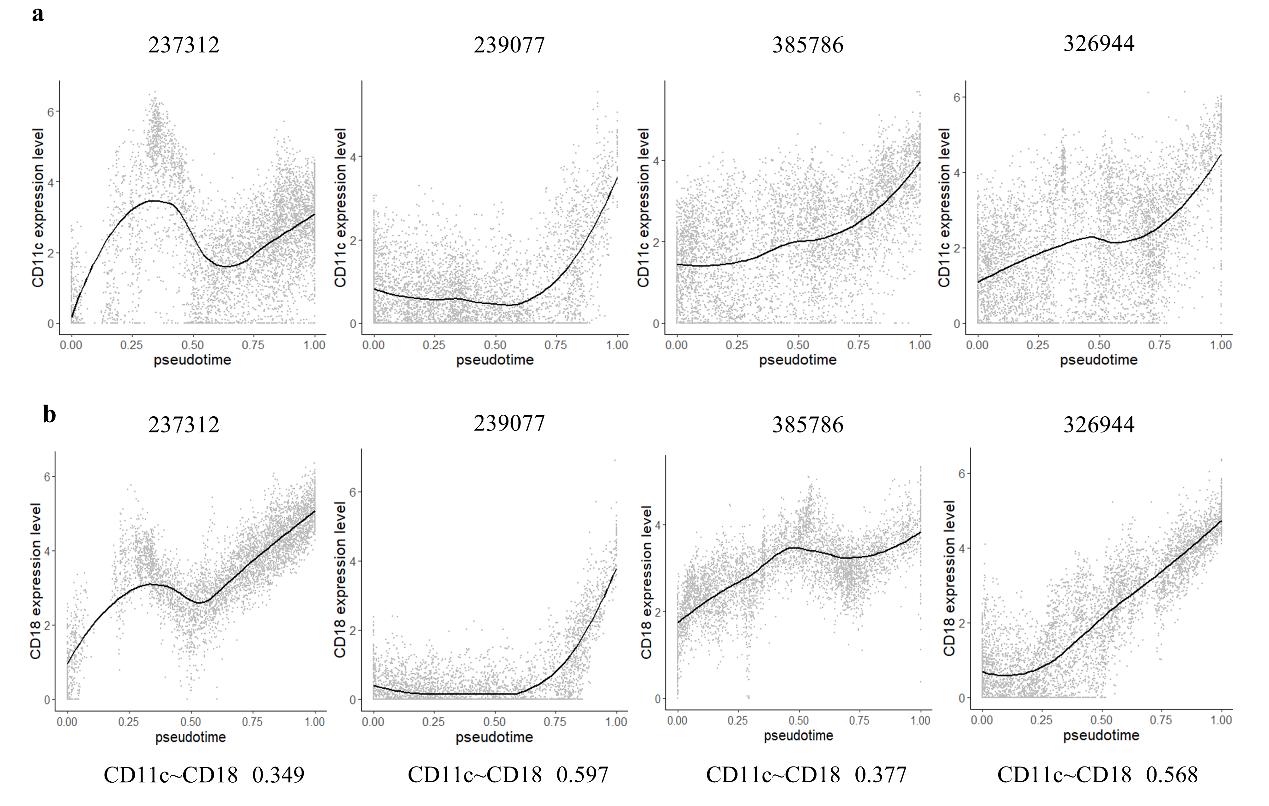


**Figure S5. The expression correlation between CD11c and CD18.** The co-occurrence of CD11c and CD18 were presented on the trajectory in four CR (CD11c^+^) samples (a and b), the Spearman correlation coefficient of each sample was performed (b).

**
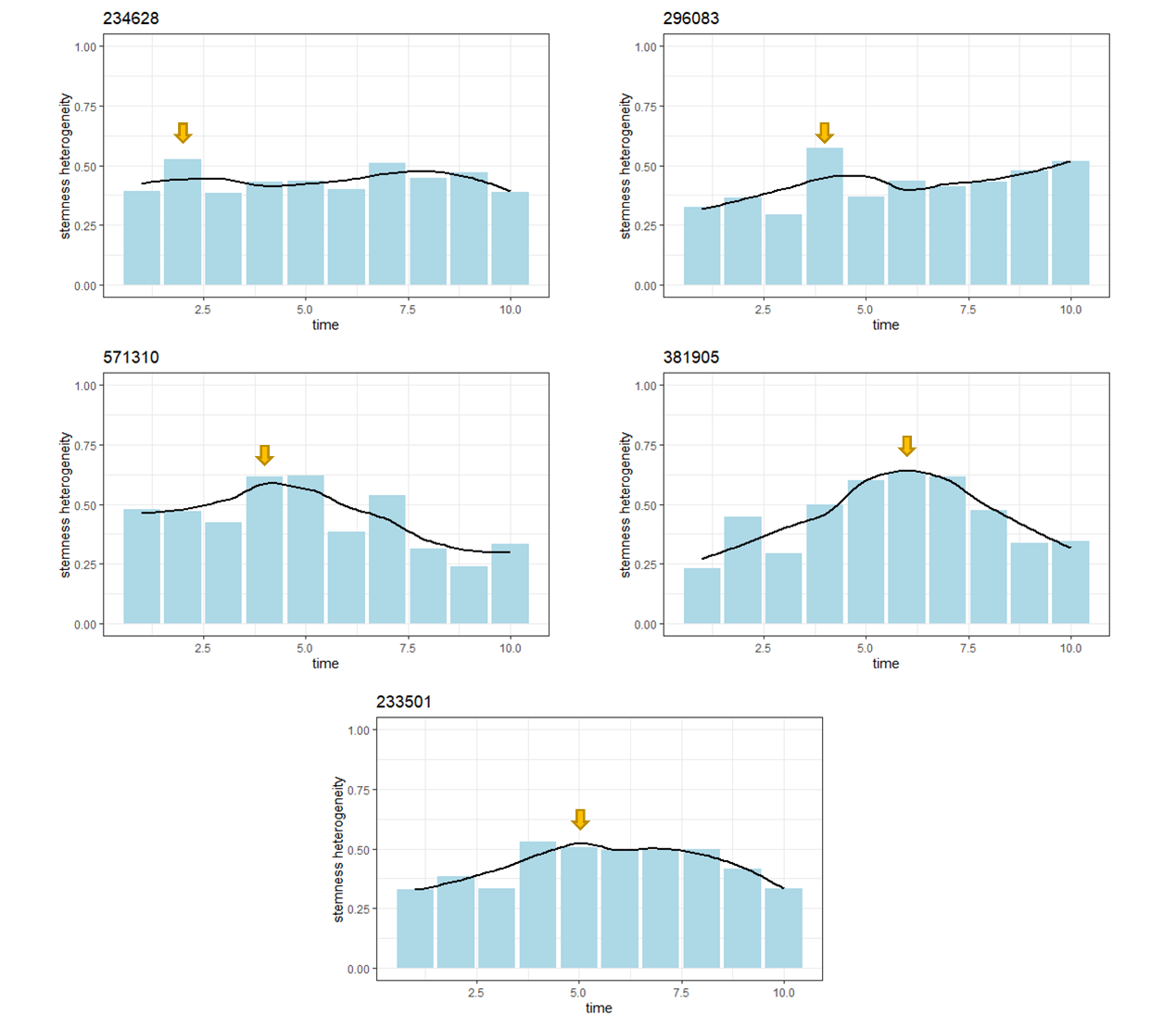
**

**Figure S6. Stemness heterogeneity in five relapse samples.** The dynamic of stemness heterogeneity was fitted by LOESS and the peak timepoint was labelled by yellow arrow.
